# A pictorial essay of thoracic wall diseases: multiple pathologies in the same anatomical site

**DOI:** 10.1186/s13244-025-02073-8

**Published:** 2025-09-20

**Authors:** Giuliana La Rosa, Miriam Adorna, Letizia Antonella Mauro, Monica Pennisi, Andrea Giovanni Musumeci, Alessandra Sigona, Claudia Mattina, Giuseppe Belfiore, Pietro Valerio Foti, Antonio Basile, Stefano Palmucci

**Affiliations:** 1 Azienda Sanitaria Provinciale di Catania, P.O. “Castiglione-Prestianni”, Bronte, Catania, Italy; 2 Department of Medical Surgical Sciences and Advanced Technologies “GF Ingrassia”, Radiology Unit 1, University Hospital Policlinico “G. Rodolico-San Marco”, Catania, Italy; 3 Department of Medical Surgical Sciences and Advanced Technologies “GF Ingrassia”, UOSD IPTRA (Pulmonary and Advanced Radiological Techniques Unit) University Hospital Policlinico “G. Rodolico-San Marco”, Catania, Italy; 4 Casa di Cura “Regina Pacis”, San Cataldo, Italy; 5 Ambulatory Specialist “UOSD Radiologia Territoriale-Centro Amianto e Risonanza Magnetica Multidistrettuale” ASP 8 Siracusa, Siracusa, Italy; 6 Department of Clinical and Experimental Medicine, “Regional Referral Center for Rare Lung Diseases”, University - Hospital Policlinico “G. Rodolico- San Marco”, University of Catania, Catania,, Italy

**Keywords:** Thoracic wall, Neoplasms, Infections, Multidetector computed tomography, Magnetic resonance imaging

## Abstract

**Objectives:**

To describe diagnostic and radiological features of the main pathologies affecting the thoracic wall, providing a pictorial atlas based on several clinical cases extracted from our archive.

**Materials and methods:**

A wide variety of pathologies affect the tissues of the thoracic cage; these conditions are often encountered by radiologists during examinations performed for unrelated clinical questions. Modern imaging techniques enable the detection of these pathologies and allow definitive diagnoses to be achieved.

**Results:**

Pathological processes that involve the chest wall may be classified into: (1) congenital and developmental diseases: pectus excavatum, pectus carinatum, supernumerary rib syndrome, Poland syndrome, neurofibromatosis, osteogenesis imperfecta, mucopolysaccharidosis, Marfan syndrome; (2) infectious and inflammatory diseases—such as aspergillosis, tuberculosis, abscesses from pyogenic bacteria, Tietze’s syndrome; (3) bone injuries (traumatic and degenerative diseases): sternal, vertebral and costal fractures, degenerative disc and arthrosis pathology; (4) chest wall tumors—such as sarcomas, lymphomas, neurogenic tumors, lipoma.

**Conclusions:**

Thoracic wall pathologies include a wide spectrum of conditions, with some clinical implications that often require a correct nosological framing. Recognizing these pathologies is essential for radiologists so that they can make a correct description in the report and direct toward appropriate treatment if required.

**Critical relevance statement:**

Cage diseases are various and difficult to understand, so multimodality imaging plays a crucial role in achieving an efficient and final diagnosis.

**Key Points:**

- Thoracic wall pathologies have different etiologies.
- Imaging represents a fundamental tool to clarify their extension, location, and nature.
- The prognosis of some of these diseases can be poor.

**Graphical Abstract:**

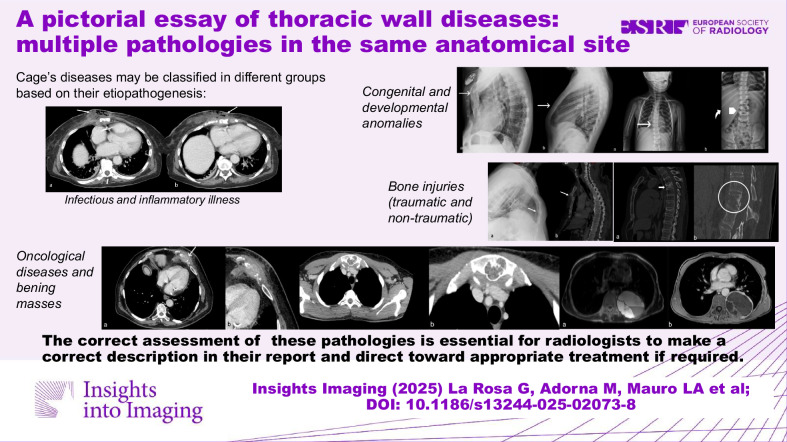

## Introduction

Thoracic wall diseases include a wide spectrum of conditions, since they may develop from different tissues, as bone structures (ribs, sternum, spine), respiratory and dorsal muscles, nerves, cartilaginous elements, and adipose tissue. Imaging often allows diagnosis, with ultrasound and X-rays useful for initial assessment. Some conditions require further evaluation with multidetector CT or MRI to better define lesion location, extent, and nature [1].

## Materials and methods

This pictorial review was conducted by retrospectively collecting thoracic wall disease cases from our institutional radiology database and from the clinical archives of our tertiary care center between June 2018 and February 2024. We selected representative cases of congenital, inflammatory, traumatic, and neoplastic chest wall conditions based on imaging findings, clinical documentation, and confirmed diagnoses. In particular, we selected Ultrasound (US), chest Computed tomography (CT) and Magnetic resonance imaging (MRI) examinations performed with dedicated protocols suitable for musculoskeletal and soft-tissue assessment, as well as chest radiographs and ultrasound examinations when available. All imaging data were reviewed and discussed by two radiologists with more than 5 years of experience in thoracic imaging. All selected cases were anonymized and analyzed for radiological features, location, tissue of origin, and diagnostic clues relevant to differential diagnosis. Each case was categorized into predefined groups: (1) congenital and developmental disorders, (2) infectious and inflammatory diseases, (3) traumatic and degenerative injuries, and (4) chest wall tumors.

## Results

### Congenital and developmental pathologies: pectus excavatum, pectus carinatum, supernumerary rib syndrome, Poland syndrome, neurofibromatosis, osteogenesis imperfecta, mucopolysaccharidosis

#### Pectus excavatum

Pectus excavatum is the most frequent congenital sternal deformity (90% of cases) affecting males most frequently [1]. Patients are commonly asymptomatic, though some experience exertional dyspnea. Beyond aesthetic problems, it may occasionally be associated with mitral valve prolapse, impaired restrictive pulmonary function tests, and cardiac function abnormalities [2, 3]. It presents as a concave sternal depression with bilateral rib protrusion. The cause is thought to be overgrowth of the lower costal cartilages, forcing the sternum inward [3].

##### Radiological features.

**Fig. 1:**
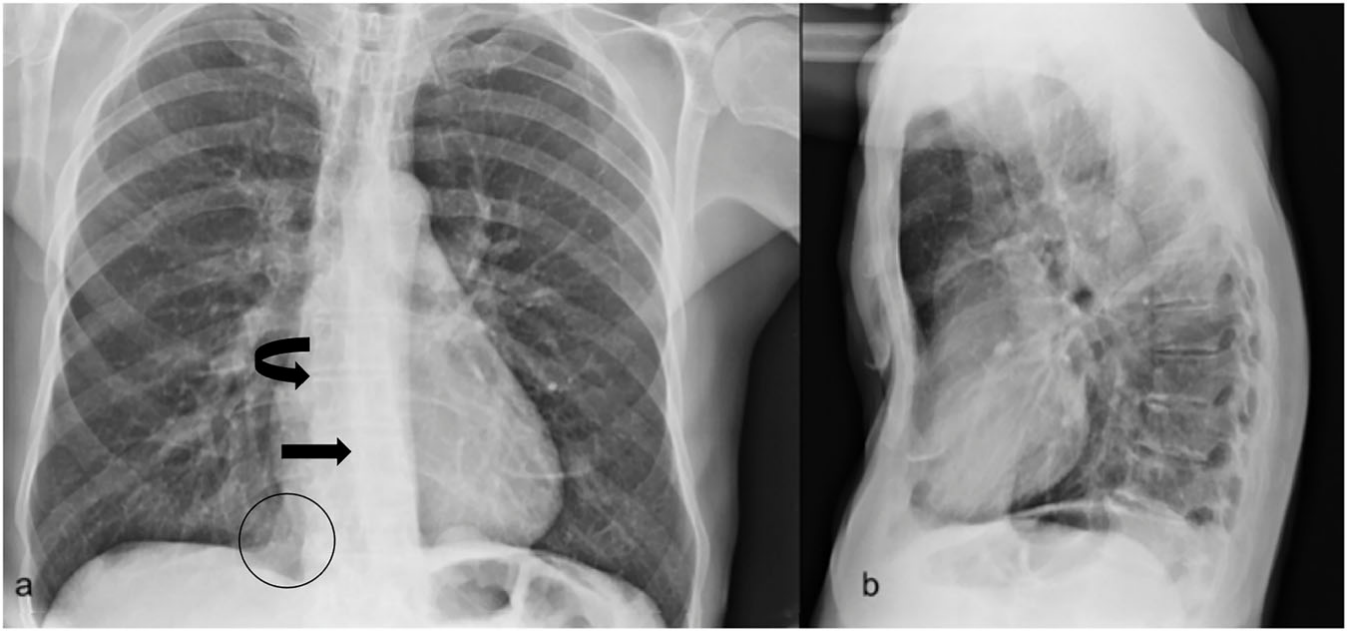
Chest X-ray was performed in orthostatism in two standard projections. This is a case of a 61-year-old male patient with a history of cardiac arrythmia. The posteroanterior projection (**a**) shows increased inferiorly-medial right hypodiaphany, related to increased visibility of the parasternal soft tissues located in the anterior chest wall (black circle), blurring of the right cardiac margin (curved black arrow) and axial rotation of heart with dislocation to the left side in the corresponding area of descending aorta (black arrow); on lateral view, the degree of depression could be easily assessed (**b**)

Posteroanterior chest radiography (X-ray) documents axial rotation of the heart with dislocation to the left side in the corresponding area of the descending aorta [4], blurring of the right cardiac margin, and an increased inferiorly-medial right hypodiaphany, related to increased visibility of the parasternal soft tissues located in the anterior chest wall (Fig. 1). In the lateral projection, it is also possible to estimate the severity of sternal depression. The tomographic investigation helps in quantifying the degree of deformity. The “pectus index,” assessing the degree of sternal dislocation, is defined as the ratio between the inner transverse diameter of the thorax and the shortest internal anteroposterior distance between the posterior aspect of the sternum and the anterior surface of the vertebral body, typically measured on axial CT scans at the point of greatest sternal depression (Fig. 1s, electronic supplementary material). Haller et al have defined a normal value of this index as around 2.56; values above 3.25 have been correlated with surgical treatment. Other parameters assessed on CT imaging (Fig. 1s, electronic supplementary material), such as the degree of depression, thoracic asymmetry, and flattening of the chest, are crucial in predicting surgical outcomes [5]. The differential diagnoses include areas of atelectasis, consolidations of the middle and/or left lower lobe, heterologous tissue in the para-aortic region, mediastinal mass, and pulmonary or paravertebral mass.

#### Pectus carinatum

Pectus carinatum—otherwise known as “pigeon chest” refers to an abnormal anterior sternal protrusion and may be associated with congenital cardiac abnormalities. Patients can manifest respiratory functional alteration. It can also be associated with other skeletal disorders, like scoliosis or Marfan syndrome.

##### Radiological features.

**Fig. 2:**
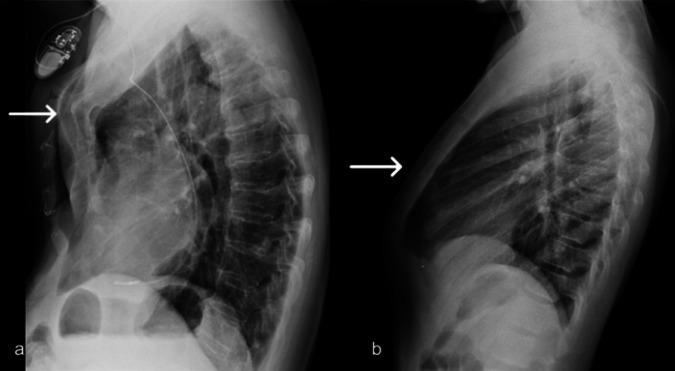
Pectus carinatum. Lateral chest X-ray views demonstrate the two types of sternal protrusion: chondromanubrial with protrusion of the manubrium and upper sternal portion (**a**), and chondrogladiolar with protrusion of the median and inferior region of the sternum (**b**)

Diagnosis is allowed thanks to lateral X-ray projection (Fig. 2) or CT imaging (axial and multiplanar reconstruction imaging in Fig. 2s). There are two types of sternal protrusion: chondrogladiolar with protrusion of the median and inferior region of the sternum, and chondromanubrial with protrusion of the manubrium and upper sternal portion (less common). This last is also called Currarino-Silverman syndrome [6, 7].

#### Supernumerary rib syndrome

Also known as accessory or “Eve’s rib,” the supernumerary rib usually originates from the seventh cervical vertebra and articulates with a transverse cervical process. Occurring in 0.5% of the population, it is often asymptomatic [8]. It varies in size and is distinguished from the second true rib at the manubriosternal joint [8]. Diagnosis is made on imaging, although it may be supported by symptoms such as ipsilateral arm pain, edema, or altered pulses. Supernumerary ribs may cause thoracic outlet syndrome with vascular and neurogenic compression, including numbness, paresthesia, or weakness in the upper limb [9].

##### Radiological features.

**Fig. 3:**
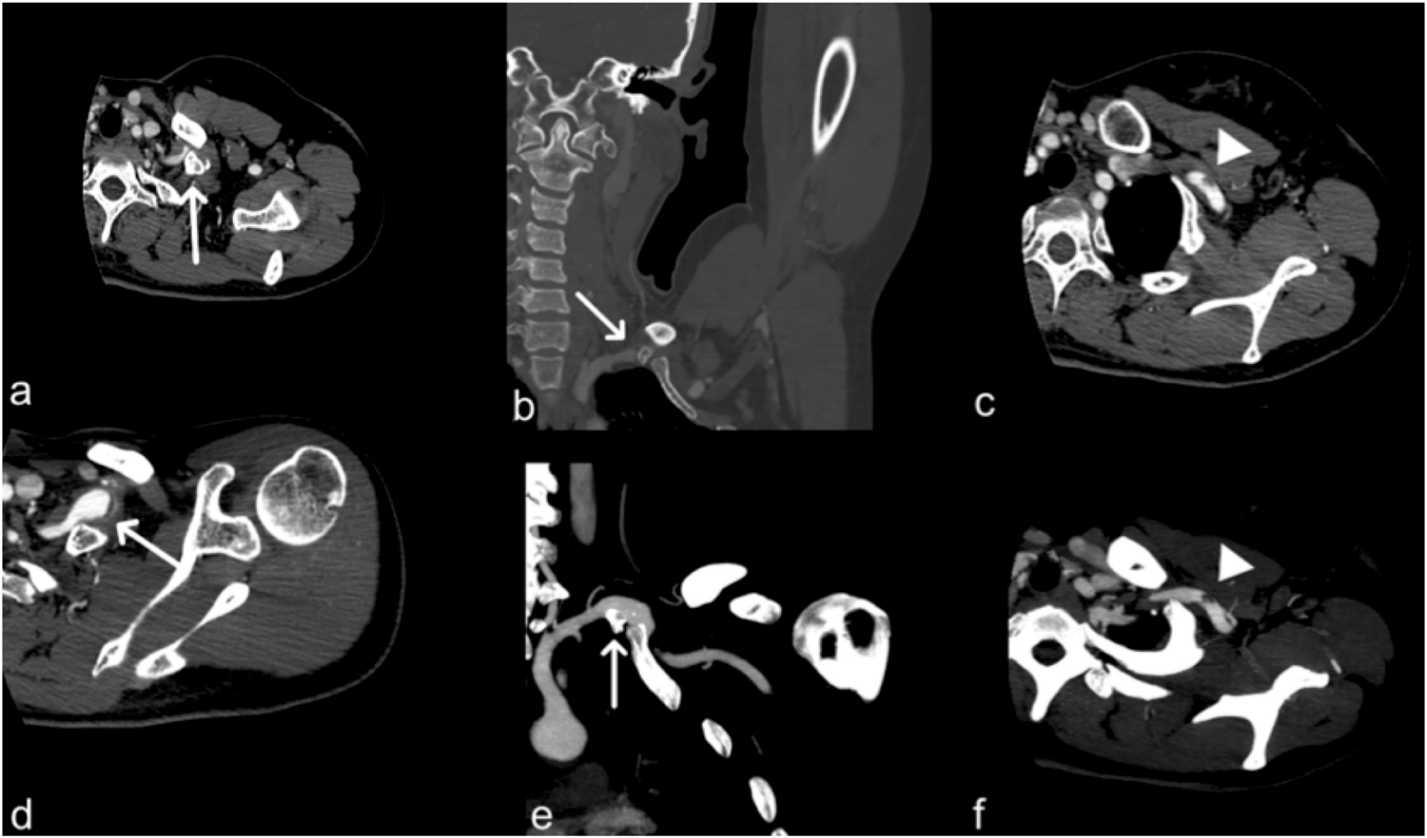
A 35-year-old female patient with a supernumerary rib. CT images after contrast medium injection, in arterial phases (**a**–**d**) and the same phase after maximum intensity projections reconstruction (**e**, **f**). They show the supernumerary rib, which causes the development of a thoracic outlet syndrome (white arrows in **a**—axial view and soft tissue window, and **b**—coronal view with bone window). **c** and **f** also demonstrate partial thrombosis of the subclavian artery (white arrowheads)

Ultrasound and tomographic imaging are performed with the patient’s arms in neutral (adducted) and elevated (abducted) positions (Fig. 3). MRI is useful for assessing the involvement of neurogenic structures as the edema surrounding the brachial plexus and the loss of surrounding fatty tissue. Venous involvement may consist of subclavian-axillary venous thrombosis, collateral compensation circles, stenosis, and narrowing of the venous vessel. Arterial involvement consists of axillary-subclavian aneurysms or pseudoaneurysms, arterial thrombus, distal emboli, collaterals, and vessel narrowing [10].

#### Poland syndrome

This is a rare congenital anomaly characterized by total or partial absence of the pectoralis major and ipsilateral syndactyly, often associated with, absence or atrophy of ribs, pectoralis minor, and nipple or breast aplasia [11, 12].

##### Radiological features.

**Fig. 4:**
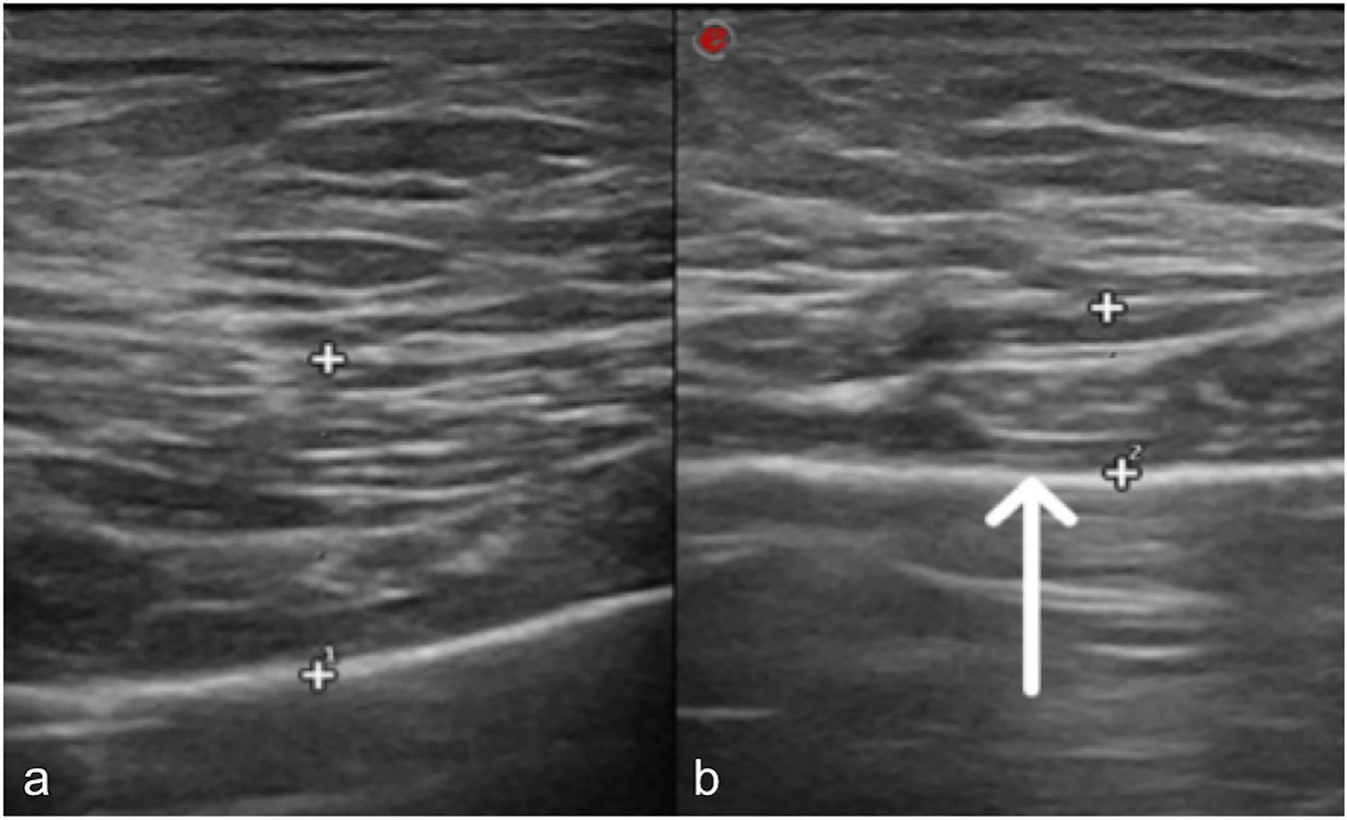
A 12-year-old male affected by Poland syndrome. Ultrasound images reveal normal pectoral muscle on the right side (**a**) and left pectoral muscle atrophy (white arrow in **b**)

Chest X-ray shows hypodiaphany emulating a mastectomy [13]. CT scan defines better muscle abnormality and associated musculoskeletal defects, while ultrasound estimates the degree of muscle atrophy (Fig. 4). Finally, mammography may help in differential diagnosis [14].

#### Mucopolysaccharidosis

Mucopolysaccharidosis represents a heterogeneous group of hereditary lysosomal storage diseases, resulting in progressive tissue damage. Symptoms include multiple dysostosis, mental retardation, and developmental delay.

##### Radiological features.

**Fig. 5:**
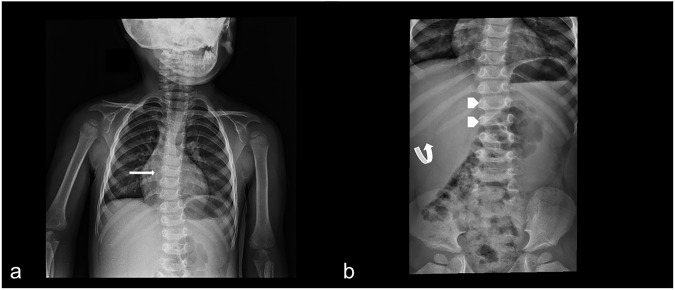
X-ray examination, spine and thorax studies. A pediatric patient with mental and developmental illness, suffering from mucopolysaccharidoses. **a** A scoliotic attitude of the thoracolumbar spine is well depicted (white arrow). Widened and extended-looking ribs, especially the lower one, could be observed in **a** and **b** (curved arrow in **b**). Vertebral bodies are rounded and flattened (white arrowhead)

The ribs present a “paddle” or “oar” appearance, with wider anterior arches and thinner posterior ones. Other rarer abnormalities are small scapulae, flattening of the glenoid cavities, small sternum, and thickened clavicles [15]. The vertebral column is frequently involved in this process (Fig. 5), and complications, like compression of the spinal cord and emerging nerves, can occur (Fig. 3s). Vertebral bodies are often rounded and flattened ‘bullet-shaped vertebrae.’ Thoracolumbar vertebrae show prolongation of the posteroinferior angle and reduction of the anterosuperior one, creating a ‘beak anterior’ appearance in the lateral x-ray. Hypoplasia of both anterior margins leads to wedge-shaped vertebrae (“platyspondyly”). These alterations can evolve into hump-like deformities, particularly in type I. Scoliosis is usually mild, but myelopathy may require surgery [16].

#### Osteogenesis imperfecta

This is a congenital disorder caused by abnormal type I collagen production, leading to fragile, osteoporotic bones. Eight different types have been reported, the mild type (I), perinatal type (II), and progressive deforming type (III) being the most frequent [17, 18].

##### Radiological features.

**Fig. 6:**
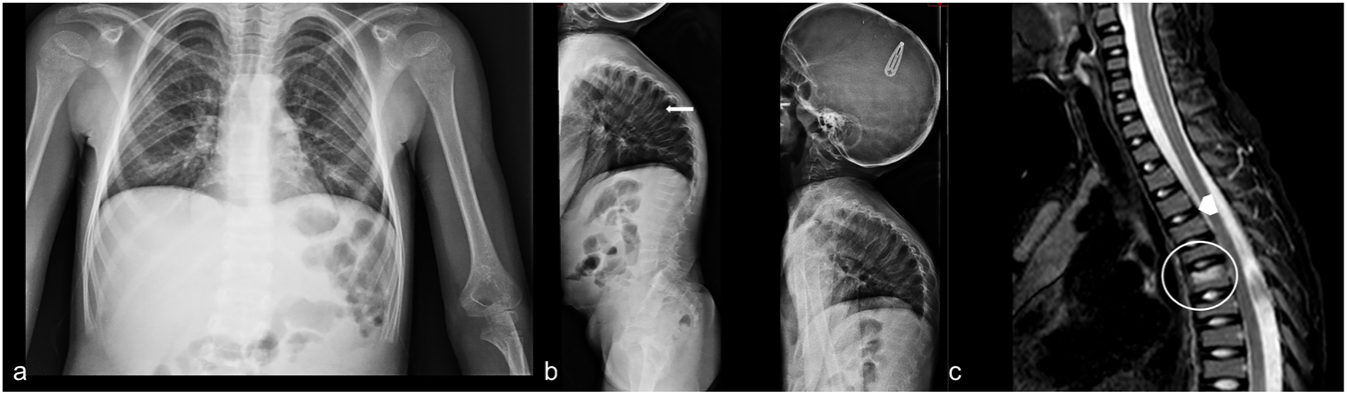
Patient affected by osteogenesis imperfecta. X-ray pictures show diffuse alteration of the vertebral bodies with a reduction in height (platyspondyly) and depression of the upper side of vertebral bodies—namely at the thoracic level (white arrow, **b**); marked gibbous deformation of the dorsal spine (**b**). Mildly dysmorphic appearance of the rib cage (**a**). MRI shows an area of signal hyperintensity in T2/STIR of the soma of Th6 due to edema (possible hyperemia or microfracture) (**c**, white circle). The intervertebral discs take an oval appearance in the absence of significant protrusions (**c**, white arrowhead)

X-rays and CT findings are vertebral compression fractures, kyphoscoliosis, a vertebra with a biconcave appearance (“codfish vertebra”), excavated or keeled chest, accordion ribs, popcorn bone calcifications, osteoarthritis, bones with a metaphyseal zebra-stripe appearance (after treatment with bisphosphonates), hyperplastic bone callus formation, cortical thickening, bone deformities (Fig. 6) [18, 19].

### Infectious and inflammatory diseases: tuberculosis, abscesses from pyogenic bacteria, Tietze’s syndrome

#### Tuberculosis (TBC)

Chest wall involvement represents a rare manifestation of TBC [20–22]. It may be caused by the contiguous spread of subpleural lesions or by hematogenous spread [23, 24].

##### Radiological features.

On CT images, the typical findings are bone and cartilage destruction, masses with calcifications, and rim enhancement [24]. Vertebral infection (Pott’s disease) typically spreads anteriorly through the anterior longitudinal ligament, sparing the posterior elements, often involving multiple levels. The vertebral body presents a reduction in height and a reduction of the anterior vertebral margin. Paravertebral abscess collections may eventually appear [25].

#### Pyogenic abscesses

The most commonly implicated organisms are Staphylococcus aureus and Pseudomonas aeruginosa [26]. Generally, osteomyelitis of the sternum, ribs, or vertebrae is associated with adjacent tissue masses, loss of subcutaneous planes, and periosteal reaction [27].

##### Radiological features.

CT and MRI can easily detect the infected tissue surrounding the bony structures, showing rim enhancement, and evaluate the adjacent pulmonary, mediastinal, and pleural structures [6, 7]. CT is preferred, especially for biopsy or drainage, and may reveal intralesional gas (Fig. 4s) [28].

#### Tietze’s syndrome

It is a benign pathology, frequently occurring between the second and fifth decade of life, characterized by a self-limiting inflammation of the costal cartilage accompanied by bone hypertrophy and reaction [29].

##### Radiological features.

**Fig. 7:**
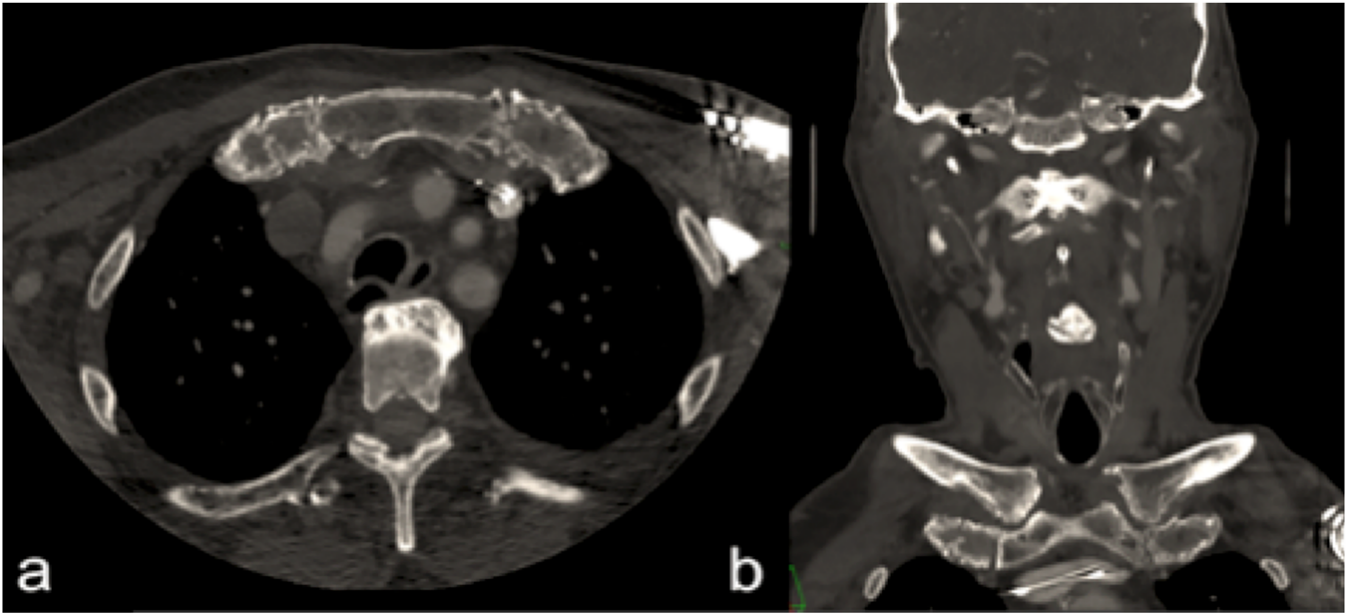
Typical case of Tietze syndrome (CT images, bone view): young man patient 30 years old, with costal pain and no history of inflammatory condition, autoimmune or neoplastic condition. Tietze syndrome is a benign condition characterized by an inflammation of the costosternal cartilage, often with hypertrophy. **a** Axial CT image (bone window) shows focal swelling and sclerosis of the second costosternal joint, with surrounding soft tissue thickening. **b** Coronal reformatted CT image confirms enlargement and sclerosis of the costal cartilage at the same level, consistent with Tietze syndrome

Ultrasound imaging shows an increase in the echogenicity of the affected cartilages and their thickness, compared to the contralateral cartilages [30, 31]. CT changes can be classified into three types: normal anatomy, focal cartilage enlargement, and ventral cartilage angulation. Other CT findings are: fracture line-like appearance corresponding to damaged cartilage, cartilage calcifications, bone sclerosis, and swelling of the underlying soft tissue (Fig. 7) [32]. The most accurate investigation is MRI, typical findings are: cartilage enlargement and thickening at the articular site on T2-weighted or STIR (Short Tau Inversion Recovery) sequences, subchondral bone-marrow edema, intense enhancement after gadolinium administration in the areas of cartilage thickening, in the subchondral bone or even in the joint capsule or ligaments. The main differential diagnosis is with costochondritis, in which the hypertrophic appearance is absent and often more than one joint is involved [29].

#### Thoracic actinomycosis

Actinomycosis, caused by gram-positive anaerobes, typically spreads from lung infections to the chest wall or through direct extension after trauma, surgery, or infection [33, 34]. Chest wall involvement is now rare due to antibiotics, but may require prolonged therapy or surgeries [35].

##### Radiological features.

On imaging, it appears as a chest wall soft-tissue mass linked to pulmonary consolidations, with low-attenuation areas, empyema, rib or vertebral destruction, and possible bronchocutaneous or intercostal fistulas [35].

### Traumatic and degenerative bone injuries: sternal, vertebral, and costal fractures, degenerative disc and arthrosis pathology

#### Rib fractures

Rib fractures, often from trauma, may be hard to detect and can be associated with pneumothorax, hemothorax, organ injury, or vascular damage. Fractures between the fourth and tenth ribs are most common; multiple fractures can cause a “floating rib” or “coastal volet” [36–38].

##### Radiological features.

Complete fractures are well detected on the chest radiograph, even though CT is more sensitive (Fig. 5s) [36–38]. Ultrasonography can be useful in assessing possible complications, such as pneumothorax or associated pulmonary contusion [39, 40].

#### Sternal fractures

Sternal fractures, accounting for 5% of thoracic injuries, mainly affect the manubrium and may result from trauma, motorbike accidents, or cardiopulmonary resuscitation. They often associate with rib fractures, joint dislocation, or mediastinal injuries. They can be distinguished into direct from trauma or indirect from deceleration.

##### Radiological features.

Sternal fractures appear as hypodiaphany on X-ray (Fig. 6s-a), better seen with sternal-lateral views. CT is the most accurate method to detect cortical breaches and associated injuries [41, 42] (Fig. 6s-b).

#### Vertebral fractures

Vertebral fractures of the thoracic tract are often the result of blunt trauma such as motor vehicle accidents [43]. The thoracic vertebral tract, compared to the lumbar, is stiffer and requires more energy to cause trauma. The thoracolumbar junction is the most frequent fracture zone as a result of the high mobility of this tract [44].

##### Radiological features.

Vertebral fractures include compression, burst, posterior column, flexion-distraction (seatbelt- “Chance Fractures”), fracture-luxation, translation-rotation, and transverse process fractures [44, 45]. Classifications include AO, Thoracolumbar Injury Classification and Severity score (TLICS), and Genant [46–48]. Elements that lean toward the neoplastic form are: total bone destruction visible on CT scan, signal alterations in the vertebral soma on MRI, epidural or paraspinal masses, concomitant vertebral metastases, bulging of the vertebral cortical bone in the canal with involvement of the medullary canal (Fig. 7s) [49, 50].

#### Degenerative disc disease and arthrosis

Arthrosis is a widespread pathology with high morbidity and social costs [50, 51]. It is classified into: primary (idiopathic) and secondary (post-traumatic, post-surgical, occupational stress, obesity, etc.) [52].

##### Radiological features.

The main radiographic features are joint space narrowing, subchondral sclerosis, and osteophytosis. Others include: joint space narrowing (asymmetrical), joint erosion (typical of the acromioclavicular joint in the thoracic location), subchondral cysts, bone marrow lesions visible on MRI as edema appearance, and synovitis [53–55].

Degenerative disc disease is related to biomechanical stresses and genetic predisposition, which alter the metabolic and structural integrity of the intervertebral disc [56].

##### Radiological features.

Disc abnormalities are subdivided into: bulging of the annulus and herniation with displacement of intervertebral disc material beyond the disc edges (protrusion, extrusion or sequestration). [57]. For the study of the spine, MRI remains the gold standard with sagittal T1 fast spin-echo (FSE), sagittal and axial T2 STIR sequences with T1-weighting and suppression of adipose tissue (in sagittal projection) and T2 gradient echo sequences (axial) [58].

### Chest wall tumors

Chest wall tumors may develop from lymphatic tissues, fat, nerves, vessels, cartilage, and bone. They have been classified as primary and secondary chest wall tumors [59, 60]. Chest wall tumors have also been classified as malignant lesions, which include mainly sarcomas (chondrosarcoma in adults and Ewing’s sarcoma among adolescents), and benign neoplasms, mostly represented by chondroma and lipoma.

#### Sarcomas

Chest wall sarcomas consist of a wide range of lesions, depending on the type of tissues from which they originate (soft tissues, muscle, bone); they include:

- Soft-tissue sarcoma: liposarcoma, fibrosarcoma, malignant fibrohistiocytoma (rapidly growing mass that can invade surrounding tissue), rhabdomyosarcoma, leiomyosarcoma and synovial sarcoma.
- Bone sarcoma: chondrosarcoma, osteosarcoma and Ewing’s sarcoma.
- Vascular sarcoma: angiosarcoma (often presents as a bruised or swollen area that may bleed easily).

They may be incidentally encountered on imaging [61, 62]. Sometimes they are metastases, such as myxoid liposarcomas, which can spread to the chest wall [63]. In a recent series of 57 chest wall sarcomas published by Gangopadhyay, 30 were soft tissues and 27 were bone sarcomas [64]; among osseous and cartilaginous tumors, chondrosarcomas represent the most primary sarcoma encountered in the chest wall [60]. According to the paper by Czarny et al, soft-tissue sarcomas represent less than 1% of incident solid tumors yearly [65].

##### Radiological features.

Liposarcoma can develop in the fatty tissues of the chest wall. It often presents a slowly growing, is painless, but larger tumors can cause discomfort or pressure symptoms.

The most common types of liposarcoma are represented by well-differentiated, dedifferentiated, myxoid, pleomorphic, and mixed lesions.

Myxoid liposarcomas show fluid-like signals with high intensity on T2-weighted images in MRI studies. Calcifications and ossifications can be seen. The pleomorphic liposarcoma is the most aggressive type, and it is difficult to define because of its heterogeneous appearance [66, 67].

**Fig. 8:**
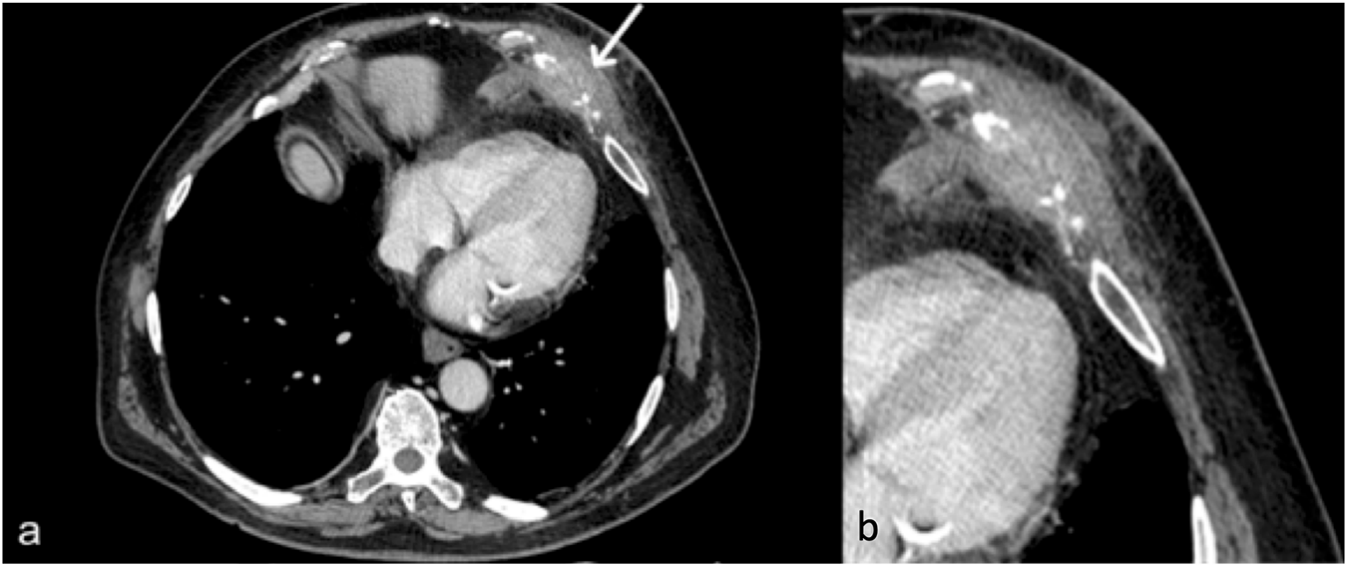
Rib chondrosarcoma in a 30-year-old male patient. **a** Axial contrast-enhanced CT image (venous phase) shows a soft tissue mass originating from the left costal cartilage with adjacent chondral erosion (white arrow), without extension into the underlying muscle layers. **b** Sagittal reconstruction confirms the cartilaginous origin and the absence of deep muscle invasion

Chondrosarcoma appears as lobulated expansive masses—typically centered on ribs (Fig. 8), but it could also be stuck sternum or scapula (shoulder blade). On CT, it shows calcifications with arch or dense or stippled appearance; heterogeneous enhancement could be detected. On MRI, the typical aspects are: high signal intensity lesions on T2-weighted images, with some low signal areas (may indicate dense mineralization) [62–68]. Concerning chest wall osteosarcomas, they usually arise in the ribs. They can cause pain, swelling, and sometimes a visible mass. Because they involve bone, it may also weaken the ribs, leading to fractures.

Periosteal osteosarcoma involves the cortex externally and typically shows irregular or lobulated margins despite the cortical bone. CT and MRI investigations are useful in assessing bone-marrow involvement [69]. Ewing’s sarcomas of the chest wall are malignant tumors that occur in pediatric or young adult patients, typically between 10 and 20 years of age, and are related to 11,22 chromosomal translocation. One of the most common symptoms is localized chest pain, often worsening with deep breathing or physical activity [70]. Ewing sarcoma, specifically affecting the chest wall, is defined as Askin tumor. The imaging appearance is characterized by heterogeneous enhancement, eccentric growth, and rare calcifications. The common sites for Ewing sarcoma in the chest wall include the ribs, sternum, costal cartilage, and thoracic spine. Nonetheless, it can also affect the intercostal muscle and other soft tissue [71]. It appears isointense to the muscle on T1-weighted images and usually shows high signal intensity on T2 sequences [62].

#### Neurogenic tumors

Neurogenic tumors—such as neuroblastoma, ganglioneuroblastoma, ganglioneuroma, neurinoma, neurofibroma, neurofibrosarcoma—may arise from the intercostal nerves or the paraspinal ganglia of the sympathetic nerve system chain.

##### Radiological features.

**Fig. 9:**
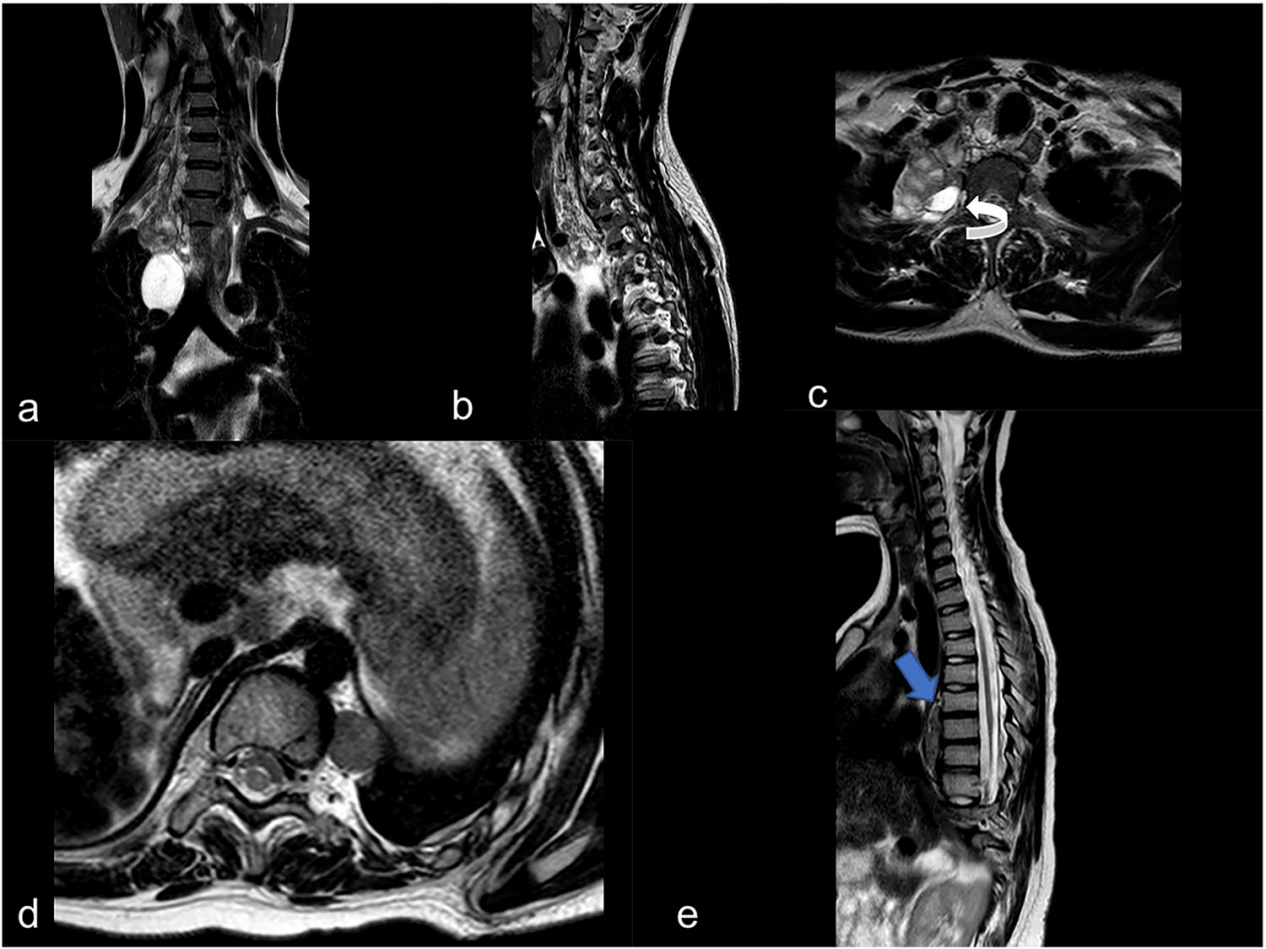
MRI-T2 sequences with fat suppression. **a**–**c** Neurofibroma arising from the neck and extending to the upper thoracic site, (**c**) widened right neural exit foramina (white curved arrow). **d**, **e** A typical case of pediatric paravertebral neurinoma with a rounded aspect (**d**). In this case, lesions do not cause bone erosion but vertebral scalloping in the thoracic vertebral body at the level of Th5 (blue arrow). Both tumors show shade hyperintense aspects on T2-weighted sequences

They show high signal intensity on T2-weighted sequences, with a fusiform appearance. Neurofibromas can occur anywhere along peripheral nerves and often involve multiple nerve fascicles, leading to a fusiform shape (spindle-shaped), are usually isointense or hypointense on T1-weighted images and typically hyperintense on T2-weighted images compared to muscle (Fig. 9a–c). Often these tumors show a “target sign” with a typical central area of the lesion lower in signal (due to dense collagen or fibrous tissue), surrounded by a higher signal rim (due to myxoid tissue or less dense cellular areas). Finally, neurofibromas usually show heterogeneous enhancement (though some may enhance uniformly). In patients with neurofibromatosis type 1 (NF1), plexiform neurofibromas appear as a complex, “bag of worms” or “serpentine” mass on imaging, often with an extensive and irregular pattern of enhancement [62, 72].

**Fig. 10:**
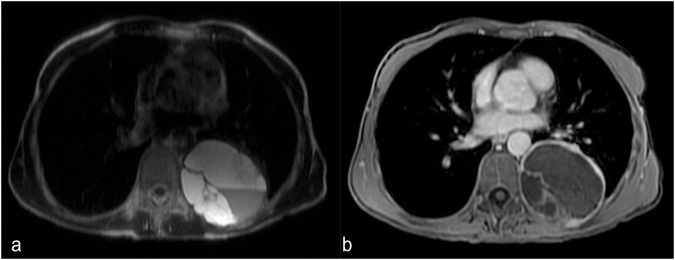
Magnetic resonance is a promising imaging modality to confirm neurogenic tumors (like paragangliomas)—the internal fluid produces a hyperintense signal on T2-weighted MRI images (**a**). **b** A typical hypointense aspect on the T1-weighted image

Neurofibromas may be found in the posterior mediastinum (paravertebral region), in the cardiac region (Fig. 8s), at the cervicothoracic region (Fig. 9s), or along the course of the cutaneous nerve (Fig. 10s).

Paragangliomas are rare tumors that arise from the paraganglia, small collections of neuroendocrine cells associated with the autonomic nervous system. When they occur in the chest wall, they are usually associated with the sympathetic chain. Functional paragangliomas can cause symptoms such as hypertension, palpitations, and headaches due to excess hormone secretion [62]. On CT, paragangliomas usually appear as well-defined, heterogeneously enhancing soft-tissue masses. They may have a lobulated appearance a small calcification within. Bone erosion could be assessed. On MRI, their behavior is very similar to other neurogenic tumors, and after gadolinium administration, they show a significant enhancement due to their vascularity (Fig. 10).

Neuroblastoma is more frequent intra-abdominally than thoracic (retroperitoneal, paravertebral), in the chest wall often arises from the sympathetic chain, typically in the posterior mediastinum (the area behind the heart and between the lungs). It can extend into the chest wall muscles or adjacent structures. Typically, it is found in pediatric age or early adulthood. This tumor manifests itself as a mass in the chest cavity or within the chest wall, potentially involving the ribs, spinal column, or paraspinal region, and may or may not be accompanied by pain or other symptoms related to its activity. It presents a heterogeneous appearance with calcifications and necrosis in 80–90% of cases; on MRI images, it shows heterogeneous signal intensity (but usually low on T1 imaging and high on T2 sequences) [73]. The extent and localization of tumors originating from nerves are easily studied with MRI; sometimes it can involve pleural space [62, 72].

Malignant peripheral nerve sheath tumors (MPNST) are neoplasms of nerve that both infiltrate locally and metastasize. The mean age of incidence is 42 years old and it is related to type 1 neurofibromatosis [74]. The internal architecture of the tumor may be heterogeneous. On T2-MRI imaging, it shows signal intensity markedly increased [75]; malignant transformation usually is associated with loss of target-like appearance on T2 images [72].

#### Metastases

Metastases are related to the spreading of primary malignant lesions—by the lymphatic or hematogenous route—along the thoracic wall.

##### Radiological features.

Ribs, vertebral bodies and sternum may be interested by metastases, some of them reproducing specific imaging features of primary malignancies from which they are derived: sclerotic lesions from breast or prostate cancer, lytic from renal carcinoma, ma or multiple myeloma [76]. Soft-tissue metastatic disease is seen in extensive body involvement and it is associated with poor prognosis [77].

Chest wall metastases could involve the cage directly from adjacent thoracic tumors or superior/anterior mediastinal compartments, as frequently observed in cases of breast cancer, lung carcinoma, mesothelioma, thymic carcinoma, and lymphoma.

**Fig. 11:**
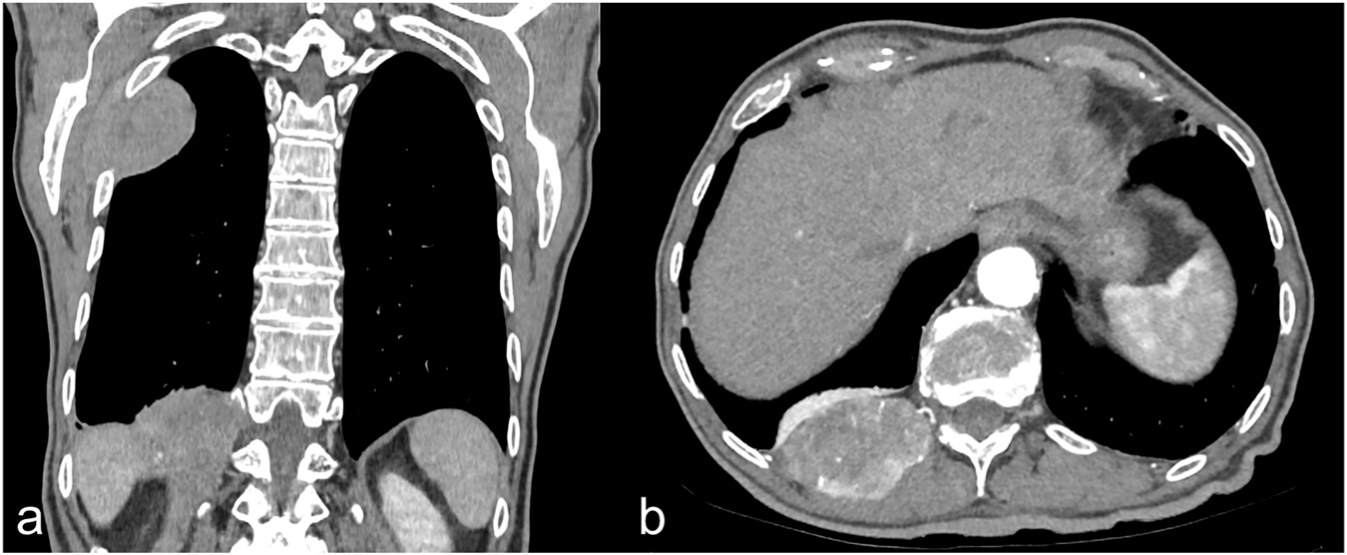
Secondary rib lesions from hepatocellular carcinoma in a patient with known liver malignancy. **a** Coronal CT image in the basal phase shows an oval-shaped, expansile lesion invading the rib. **b** Axial CT image in the arterial phase demonstrates the hypervascularization of the lesion, consistent with secondary spread of hepatocellular carcinoma

Ultrasound and CT imaging could be useful to detect lesions of unknown origin (Fig. S9). Magnetic resonance with cine sequences could be performed to confirm the presence of invasion and pleural sliding [78]. PET-CT is a valid technique for staging thanks to the measurement of increased rate of glucose metabolism and oxygen consumption in secondary tumor lesions [79]. For example, in a case report by Yang et al [80], PET-CT was also useful to detect secondary locations of hepatocellular carcinoma; in this case, primary lesions were difficult to detect by the only CT evaluation. Another case report by Godar et al deals with secondary chest wall location of hepatocellular carcinoma [81]. In this last case, like in our case, it involved ribs with erosion of the cortex. Inferiorly, it abuts the left hemidiaphragm, while posteriorly it abuts the serratus anterior and latissimus dorsi muscle without invasion (Fig. 11).

#### Enchondromas

These represent benign tumors of the intramedullary cartilage; chest wall enchondromas may involve ribs, sternum, and scapula.

##### Radiological features.

**Fig. 12:**
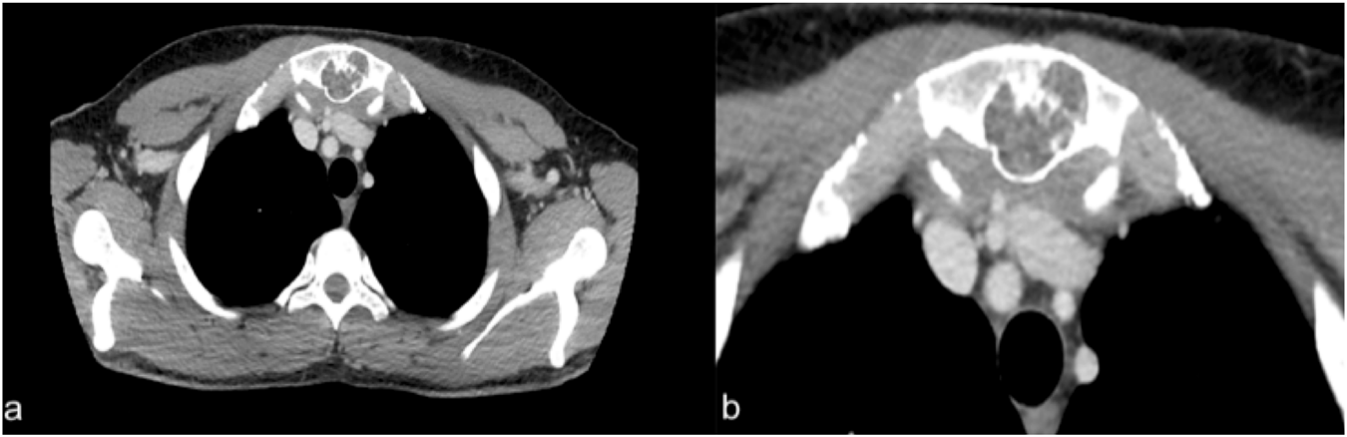
Sternal enchondroma in a 66-year-old male patient. **a** Axial CT image in mediastinal window shows a lytic lesion with defined margins involving the manubrium sterni. **b** More cranial axial CT slice confirms the cartilaginous nature of the lesion, with absence of aggressive features such as soft tissue extension or cortical disruption

They typically consist of lesions < 5 cm, lytic, with a non-aggressive appearance and defined margins [82] (Fig. 12). They may present calcifications and, unlike malignant forms, lack bone destruction, periosteal reaction, or surrounding tissue mass [82].

#### Lipomas

Lipomas are the most frequent benign chest wall tumors [83]; they may be classified as superficial or deep, depending on the subcutaneous or intramuscular location [83]. The tumor does not require any treatment as long as they are small in size.

##### Radiological features.

CT and MRI show these masses with well-defined and circumscribed margins of purely adipose consistency (Fig. 11s). They may have a bell-shaped appearance and can be intrathoracic; CT and/or MR are extremely useful in assessing their size, location, and relationship with adjacent structures [84]. The presence of fat tissue is easily demonstrated on MR images, using T1-T2-weighted images and fat suppression, in which the mass shows a homogeneous loss of signal intensity. Usually are homogeneous with nodular enhancement, and they may contain septa less than 2 mm in thickness or calcifications [85].

However, atypical appearances may be found on imaging, namely when lipomas exhibit a large size (more than 5 centimeters) or develop into the pleural cavity [86].

Intramuscular lipomas are a potential pitfall and may mimic liposarcoma with a typical infiltrative and striate appearance [87].

MRI has a high sensitivity to differentiate well-differentiated liposarcoma but a low predictive value [88]. Features that favor well-differentiated liposarcoma are: size > 10 cm, percentage of fat < 75%, thick or nodular septa > 2 mm, prominent high T2 signal foci, prominent enhancement, associate non-adipose mass, intramuscular location [89].

Another pitfall is lipoma variants. These lesions are neither simple lipoma nor well-differentiated liposarcoma but are made up of a mix of chondroid lipoma, osteolipoma, hibernoma, angiolipoma, lipoleiomyoma, and necrotic lipoma [87].

#### Elastofibroma dorsi

This lesion derives from the accumulation of fibrotic tissue with elastic fibers between the serratus anterior and latissimus dorsi muscles. It typically occurs in women, bilaterally in up to two-thirds of cases [90].

##### Radiological features.

On MRI and CT images, it shows similar aspects to skeletal muscle (Fig. 12s). On T2-weighted images, the lesion is mildly hyperintense, in particular on fat-suppressed MR sequences. Sometimes, it shows linear fat areas within the tumor termed “lasagna sign” because of their appearance [90]. In sporadic cases, it is located posterior first or second rib and deep into the superior scapula [91]. If the mass is large or shows atypical findings, a percutaneous biopsy must be performed. Differential diagnosis is difficult when these pseudotumors are deep to the fascia and more than 5 cm in size. Also, when lesions show a poorly circumscribed appearance, contrast enhancement, and heterogeneous aspects, the exclusion of soft-tissue sarcoma is difficult [89].

#### Fibrous dysplasia

These lesions originate from an anomaly development of normal marrow which is replaced with fibrous tissue. This can result in pathologic fracture or rarely in malignant degeneration. It could affect ribs (one of the most benign lesion at that level) usually in the lateral or posterior sites. These lesions demonstrate endosteal scalloping and cortical thickening, but the core remains intact despite malignant formations [92].

##### Radiological features.

On X-ray and CT images, it shows typical bully cyst aspect due to its ground-glass matrix. But depending on the degree of fibrous component it could be lucent or sclerotic. On MRI tends to be hypointense to muscle on T1 imaging and hypo or hyperintense on T2, and the enhancement is variable [93].

## Discussion

Chest wall pathologies encompass a wide spectrum of conditions, with some clinical implications that often require correct nosological framing. Chest X-ray is essential to detect and localize lesions; CT and MRI play a pivotal role in the diagnosis, since they better define their origin and extension. Some thoracic pathologies may reproduce typical imaging findings (such as lipomas or elastofibroma dorsi), and the diagnosis may be easily achieved. On the other hand, many lesions show indeterminate imaging features, so the diagnosis requires further investigation, such as biopsy.

## Supplementary information

ELECTRONIC SUPPLEMENTARY MATERIAL

## Data Availability

All data and materials are available at the Department of Medical Surgical Sciences and Advanced Technologies “GF Ingrassia,” Radiology Unit 1, University Hospital Policlinico “G. Rodolico-San Marco,” Catania, Italy; in particular, in our RIS-PACS system/archive. This manuscript is based on a conference abstract, considered as a preprint of a primary research manuscript. “Radiological atlas of chest wall diseases: multiple pathologies in the same anatomical site” EPOSTM Poster presented at ECR 2023 (10.26044/ecr2023/C-19081).

## Abbreviations

MRI: Magnetic resonance imaging
STIR: Short tau inversion recovery

## References

[1] Dähnert W (2007) Radiology review manual 6th ed. Lippincott Williams & Wilkins, Philadelphia, PA

[2] Kelly RE, Cash TF, Shamberger RC et al (2008) Surgical repair of pectus excavatum markedly improves body image and perceived ability for physical activity: multicenter study. Pediatrics 122:1218–1222 19047237 10.1542/peds.2007-2723

[3] Garcia VF, Seyfer AE, Graeber GM (1989) Reconstruction of congenital chest-wall deformities. Surg Clin North Am 69:1103–1118 2551052 10.1016/s0039-6109(16)44941-8

[4] Takahashi K, Sugimoto H, Ohsawa T (1992) Obliteration of the descending aortic interface in pectus excavatum: correlation with clockwise rotation of the heart. Radiology 182:825–828 1535902 10.1148/radiology.182.3.1535902

[5] Haller JA, Kramer SS, Lietman SA (1987) Use of CT scans in selection of patients for pectus excavatum surgery: a preliminary report. J Pediatr Surg 22:904–906 10.1016/s0022-3468(87)80585-7 3681619

[6] Swischuk LE, Stansberry SD (1991) Radiographic manifestations of anomalies of the chest wall. Radiol Clin North Am 29:271–277.7 1998051

[7] Anredan L, Hall P (1961) Diminished segmentation or premature ossification of sternum in congenital heart disease. Br Heart J 21:140–142 10.1136/hrt.23.2.140 PMC1017744 13683424

[8] Fisher MS (1981) Eve’s rib (letter). Radiology 140:8417280260

[9] Lagerquist LG, Tyler FH (1975) Thoracic outlet syndrome with tetany of the hands. Am J Med 59:281–284 1155483 10.1016/0002-9343(75)90364-2

[10] Stepansky F, Hecht EM, Rivera R et al (2008) Dynamic MR angiography of upper extremity vascular disease: pictorial review. Radiographics 28:e28 17967936 10.1148/radiol.e28

[11] Pearl M, Chow TF, Friedman E (1971) Poland’s syndrome. Radiology 101:619–623.11 4331568 10.1148/101.3.619

[12] Seyfer AE, Icochea R, Graeber GM (1988) Poland’s anomaly. Ann Surg 208:776–782.12 2848462 10.1097/00000658-198812000-00017 PMC1493821

[13] Stein HL (1964) Roentgen diagnosis of congenital absence of pectoralis muscles. Radiology 83:63–66 14191657 10.1148/83.1.63

[14] Samuels TH, Haider MA, Kirkbride P (1996) Poland’s syndrome: a mammographic presentation. AJR Am J Roentgenol 166:347–348 8553944 10.2214/ajr.166.2.8553944

[15] Palmucci S, Attinà G, Lanza ML et al (2013) Imaging findings of mucopolysaccharidoses: a pictorial review. Insights Imaging 4:443–459 23645566 10.1007/s13244-013-0246-8 PMC3731470

[16] White KK, Harmatz P (2010) Orthopedic management of mucopolysaccharide disease. J Pediatr Rehabil Med 3:47–56 21791829 10.3233/PRM-2010-0102

[17] Subramanian S, Anastasopoulou C, Viswanathan VK (2023) Osteogenesis imperfecta. In: StatPearls. StatPearls, Treasure Island 30725642

[18] Renaud A, Aucourt J, Weill J et al (2013) Radiographic features of osteogenesis imperfecta. Insights Imaging 4:417–429. 10.1007/s13244-013-0258-4 23686748 10.1007/s13244-013-0258-4 PMC3731461

[19] Gazzotti S, Sassi R, Aparisi Gómez MP et al (2024) Imaging in osteogenesis imperfecta: Where we are and where we are going. Eur J Med Genet 68:104926 38369057 10.1016/j.ejmg.2024.104926

[20] Benramdane H, Nasri S, Kamaoui I, Skiker I (2024) Sternal tuberculosis: a rare manifestation of extrapulmonary disease. Radiol Case Rep 20:15–17 39429715 10.1016/j.radcr.2024.09.113 PMC11488405

[21] Iga N, Fuchimoto Y, Koyanagi T, Mizuno D, Nishi H (2021) A rare case of chest wall tuberculosis: tuberculous scapulothoracic bursitis. Respir Med Case Rep 34:10153 10.1016/j.rmcr.2021.101537 PMC8551644 34745872

[22] Saxena S, Hariharan D (2022) Extrapulmonary tuberculosis presentation in the form of a chest wall abscess with no pulmonary involvement in the UK: a case report. J Surg Case Rep 2022:rjac421 36158246 10.1093/jscr/rjac421 PMC9491861

[23] Hugosson C, Nyman RS, Brismar J, Larsson S-G, Lindahl S, Lundstedt C (1996) Imaging of tuberculosis. V. Peripheral osteoarticular and soft-tissue tuberculosis. Acta Radiol 37:512–516.18 8688232 10.1177/02841851960373P216

[24] Padley SPG, Muller NL (1993) Tuberculosis of the chest wall: CT findings. J Comput Assist Tomogr 17:271–273 8454753 10.1097/00004728-199303000-00017

[25] Burrill J, Williams CJ, Bain G et al (2007) Tuberculosis: a radiologic review. Radiographics 27:1255–1273 17848689 10.1148/rg.275065176

[26] Osinowo O, Adebo OA, Okbanjo AO (1986) Osteomyelitis of the ribs in Ibadan. Thorax 41:58–60.14 3704967 10.1136/thx.41.1.58 PMC460254

[27] Schaefer PS, Burton BS (1989) Radiographic evaluation of chest-wall lesions. Surg Clin North Am 69:911–945 2675352 10.1016/s0039-6109(16)44930-3

[28] Sharif HS, Clark DC, Aabed MY, Aideyan OA, Haddad MC, Mattsson TA (1989) MR imaging of thoracic and abdominal wall infections: comparison with other imaging procedures. AJR Am J Roentgenol 154:989–995 10.2214/ajr.154.5.2138843 2138843

[29] Rokicki W, Rokicki M, Rydel M (2018) What do we know about Tietze’s syndrome? Kardiochir Torakochirurgia Pol 15:180–182 30310397 10.5114/kitp.2018.78443 PMC6180027

[30] Martino F, D’Amore M, Angelelli G, Macarini L, Cantatore FP (1991) Echographic study of Tietze’s syndrome. Clin Rheumatol 10:2–4 2065502 10.1007/BF02208023

[31] Jurik AG, Justesen T, Graudal H (1988) Radiographic findings in patients with clinical Tietze syndrome. Skelet Radiol 16:517–523 10.1007/BF00351265 3423820

[32] Honda N, Machida K, Mamiya T et al (1989) Scintigraphic and CT findings of Tietze’s syndrome: report of a case and review of the literature. Clin Nucl Med 14:606–609 2680209 10.1097/00003072-198908000-00011

[33] Han JY, Lee KN, Lee JK et al (2013) An overview of thoracic actinomycosis: CT features. Insights Imaging 4:245–252. 10.1007/s13244-012-0205-9 23242581 10.1007/s13244-012-0205-9 PMC3609961

[34] Hsieh MJ, Liu HP, Chang JP, Chang CH (1993) Thoracic actinomycosis. Chest 104:366–370. 10.1378/chest.104.2.366 8339619 10.1378/chest.104.2.366

[35] Webb WR, Sagel SS (1982) Actinomycosis involving the chest wall: CT findings. AJR Am J Roentgenol 139:1007–1009. 10.2214/ajr.139.5.1007 6981958 10.2214/ajr.139.5.1007

[36] Bhavnagri SJ, Mohammed TL (2009) When and how to image a suspected broken rib. Clevel Clin J Med 76:309–314 10.3949/ccjm.76a.08026 19414547

[37] DeLuca SA, Rhea JT, O’Malley TO (1982) Radiographic evaluation of rib fractures. AJR Am J Roentgenol 138:91–92 6976718 10.2214/ajr.138.1.91

[38] Kaewlai R, Avery LL, Asrani AV, Novelline RA (2008) Multidetector CT of blunt thoracic trauma. Radiographics 28:1555–1570 18936021 10.1148/rg.286085510

[39] Griffith JF, Rainer TH, Ching AS, Law KL, Cocks RA, Metreweli C (1999) Sonography compared with radiography in revealing acute rib fracture. AJR Am J Roentgenol 173:1603–1609 10584808 10.2214/ajr.173.6.10584808

[40] Turk F, Kurt AB, Saglam S (2010) Evaluation by ultrasound of traumatic rib fractures missed by radiography. Emerg Radiol 17:473–477 20652719 10.1007/s10140-010-0892-9

[41] Khoriati AA, Rajakulasingam R, Shah R (2013) Sternal fractures and their management. J Emerg Trauma Shock 6:113–116 23723620 10.4103/0974-2700.110763 PMC3665058

[42] Scheyerer MJ, Zimmermann SM, Bouaicha S, Simmen HP, Wanner GA, Werner CML (2013) Location of sternal fractures as a possible marker for associated injuries. Emerg Med Int 2013:407589 24324890 10.1155/2013/407589 PMC3845240

[43] Rajasekaran S, Kanna RM, Shetty A (2015) Management of thoracolumbar spine trauma: an overview. Indian J Orthop 49:72 25593358 10.4103/0019-5413.143914 PMC4292328

[44] Khurana B, Sheehan SE, Sodickson A, Bono CM, Harris MB (2013) Traumatic thoracolumbar spine injuries: what the spine surgeon wants to know. Radiographics 33:2031–2046 24224597 10.1148/rg.337135018

[45] Atlas SW, Regenbogen V, Rogers LF, Kim KS (1986) The radiographic characterization of burst fractures of the spine. AJR Am J Roentgenol 147:575–582 3488659 10.2214/ajr.147.3.575

[46] Jiménez-Almonte JH, King JD, Luo TD, Cassidy RC, Aneja A (2018) Classifications in brief: thoracolumbar injury classification and injury severity score system. Clin Orthop Relat Res 476:1352–1358 29419629 10.1007/s11999.0000000000000088 PMC6263590

[47] Reinhold M, Audigé L, Schnake KJ, Bellabarba C, Dai LY, Oner FC (2013) AO spine injury classification system: a revision proposal for the thoracic and lumbar spine. Eur Spine J 22:2184–2201 23508335 10.1007/s00586-013-2738-0 PMC3804719

[48] Genant HK, Wu CY, van Kuijk C, Nevitt MC (1993) Vertebral fracture assessment using a semiquantitative technique. J Bone Miner Res 8:1137–1148 8237484 10.1002/jbmr.5650080915

[49] Jung HS, Jee WH, McCauley TR, Ha KY, Choi KH (2003) Discrimination of metastatic from acute osteoporotic compression spinal fractures with MR imaging. Radiographics 23:179–187 12533652 10.1148/rg.231025043

[50] Chen D, Shen J, Zhao W et al (2017) Osteoarthritis: toward a comprehensive understanding of pathological mechanism. Bone Res 5:1–13 10.1038/boneres.2016.44 PMC5240031 28149655

[51] Kloppenburg M, Berenbaum F (2020) Osteoarthritis year in review 2019: epidemiology and therapy. Osteoarthritis Cartilage 28:242–248 31945457 10.1016/j.joca.2020.01.002

[52] Sangha O (2000) Epidemiology of rheumatic diseases. Rheumatology 39:3–12 11276800 10.1093/rheumatology/39.suppl_2.3

[53] Braun H, Gold G (2012) Diagnosis of osteoarthritis: imaging. Bone 51:278–288 22155587 10.1016/j.bone.2011.11.019 PMC3306456

[54] Tanamas S, Wluka A, Pelletier J et al (2010) Bone marrow lesions in people with knee osteoarthritis predict progression of disease and joint replacement: a longitudinal study. Rheumatology 49:2413–2419 20823092 10.1093/rheumatology/keq286

[55] Hayashi D, Roemer F, Guermazi A (2016) Imaging for osteoarthritis. Ann Phys Rehabil Med 59:161–169 26797169 10.1016/j.rehab.2015.12.003

[56] Videman T, Battié M, Gill K, Manninen H, Gibbons L, Fisher L (1995) Magnetic resonance imaging findings and their relationships in the thoracic and lumbar spine. Insights into the etiopathogenesis of spinal degeneration. Spine (Phila Pa 1976) 20:928–935 7644958 10.1097/00007632-199504150-00009

[57] Costello RF, Beall DP (2007) Nomenclature and standard reporting terminology of intervertebral disk herniation. Magn Reson Imaging Clin North Am 15:167–174 10.1016/j.mric.2006.12.001 17599638

[58] Farshad-Amacker NA, Farshad M, Winklehner A, Andreisek G (2015) MR imaging of degenerative disc disease. Eur J Radiol 84:1768–1776 26094867 10.1016/j.ejrad.2015.04.002

[59] Cleveland Clinic (2022) Chest wall tumors: causes, symptoms & treatment. Available via https://my.clevelandclinic.org/health/diseases/23482-chest-wall-tumor. Accessed on: 2 June 2025

[60] Tateishi U, Gladish GW, Kusumoto M et al (2003) Chest wall tumors: radiologic findings and pathologic correlation: Part 2. Malignant tumors. Radiographics 23:1491–1508 14615560 10.1148/rg.236015527

[61] Akhtar A, Shah S, Sheikh AB et al (2017) Sarcoma arising from the chest wall: a case report. Cureus 9:1604 10.7759/cureus.1604 PMC5654960 29075582

[62] Tateishi U, Gladish GW, Kusumoto M et al (2023) Chest wall tumors: radiologic findings and pathologic correlation: Part 1. Benign tumors. Radiographics 23:1477–1490 10.1148/rg.236015526 14615559

[63] Murphey MD, Arcara LK, Fanburg-Smith J (2005) From the archives of the AFIP: imaging of musculoskeletal liposarcoma with radiologic-pathologic correlation. Radiographics 25:1371–1395 16160117 10.1148/rg.255055106

[64] Gangopadhyay A, Nandy K, Puj K et al (2021) Primary chest wall sarcoma; a single institution experience of 3 years. Cancer Treat Res Commun 27:100326 33524850 10.1016/j.ctarc.2021.100326

[65] Czarny MJ, Chow GV, Rhee DS et al (2010) Sarcoma of the chest wall: a rare tumor. Am J Med 23:7–8 10.1016/j.amjmed.2009.07.030 20103010

[66] Moch H (2020) Soft tissue and bone tumors. In: WHO classification of tumors, Vol. 3. WHO, Geneva

[67] Lee TJ, Collins J (2008) MR imaging evaluation of disorders of the chest wall. Magn Reson Imaging Clin N Am 16:355–379 18474337 10.1016/j.mric.2008.03.001

[68] Masab M, Arora E, Gupta S, Farooq H, Jindal V, Sharma S (2018) Metastatic sternal osteosarcoma: a rare tumor. Cureus 10:2206 10.7759/cureus.2206 PMC5908719 29682436

[69] Resnick D, Greenway GD (1996) Tumors and tumor-like lesions of bone. In: Resnick D (ed) Bone and joint imaging, 2nd edn. Saunders, Philadelphia, pp 991–1075

[70] Gladish GW, Sabloff BM, Munden RF, Truong MT, Erasmus JJ, Chasen MH (2002) Primary thoracic sarcomas. Radiographics 22:621–637 12006691 10.1148/radiographics.22.3.g02ma17621

[71] Saenz NC, Hass DJ, Meyers P et al (2000) Pediatric chest wall Ewing sarcoma. J Pediatr Surg 35:550–555 10.1053/jpsu.2000.0350550 10770379

[72] Levine E, Huntrakoon M, Wetzel LH (1987) Malignant nerve-sheath neoplasms in neurofibromatosis: distinction from benign tumors by using imaging techniques. AJR Am J Roentgenol 149:1059–1064 3118667 10.2214/ajr.149.5.1059

[73] Sofka CM, Semelka RC, Kelekis NL et al (1999) Magnetic resonance imaging of neuroblastoma using current techniques. Magn Reson Imaging 17:193–198 10215473 10.1016/s0730-725x(98)00102-7

[74] Sordillo PP, Helson L, Hajdu SI et al (1981) Malignant schwannoma: clinical characteristics, survival, and response to therapy. Cancer 1981:2503–2509 10.1002/1097-0142(19810515)47:10<2503::aid-cncr2820471033>3.0.co;2-3 6791802

[75] Kuhlman JE, Bouchardy L, Fishman EK, Zer-houni EA (1994) CT and MR imaging evaluation of chest wall disorders. Radiographics 14:571–595 8066273 10.1148/radiographics.14.3.8066273

[76] O’Sullivan P, O’Dwyer H, Flint J, Munk PL, Muller NL (2007) Malignant chest wall neoplasms of bone and cartilage: a pictorial review of CT and MR findings. Br J Radiol 80:678–684 16793848 10.1259/bjr/82228585

[77] Giuliano AE, Feig S, Eilber FR (1964) Changing metastatic patterns of osteosarcoma. Cancer 54:2160–2164 10.1002/1097-0142(19841115)54:10<2160::aid-cncr2820541016>3.0.co;2-p 6593114

[78] Sakai S, Murayama S, Murakami J, Hashiguchi N, Masuda K (1997) Bronchogenic carcinoma invasion of the chest wall: evaluation with dynamic cine MRI during breathing. J Comput Assist Tomogr 21:595–600 9216765 10.1097/00004728-199707000-00013

[79] Carter BW, Benveniste MF, Betancourt SL et al (2016) Imaging evaluation of malignant chest wall neoplasms. Radiographics 36:1285–1306 27494286 10.1148/rg.2016150208

[80] Yang L, Marx H, Yen Y (2011) Early finding of chest wall metastasis of hepatocellular carcinoma in a woman by fluorodeoxyglucose-positron emission tomography scan: a case report. J Med Case Rep 13:147. 10.1186/1752-1947-5-147 10.1186/1752-1947-5-147 PMC3089792 21489297

[81] Godar M (2024) Chest wall metastasis—hepatocellular carcinoma. Case study, Radiopaedia. Available via 10.53347/rID-167241. Accessed on: 2 June 2025

[82] Walden M, Murphey M, Vidal J (2008) Incidental enchondromas of the knee. AJR Am J Roentgenol 190:1611–1615 18492914 10.2214/AJR.07.2796

[83] Nicolò S, Michela T, Marco F, Marco A, Andrea G, Majed R (2019) Chest wall lipoma mimicking intrathoracic mass: imaging with surgical correlation. Radiol Case Rep 14:956–961 31193853 10.1016/j.radcr.2019.05.020 PMC6543187

[84] McTighe S, Chernev I (2014) Intramuscular lipoma: a review of the literature. Orthop Rev 6:5618 10.4081/or.2014.5618 PMC4274454 25568733

[85] Bancroft LW, Kransdorf MJ, Peterson JJ, O’Connor MI (2006) Benign fatty tumors: classification, clinical course, imaging appearance, and treatment. Skelet Radiol 35:719–733 10.1007/s00256-006-0189-y 16927086

[86] Jang HJ, Choi BH, Park SO (2021) A rare case of chest wall lipoma growing into the pleural cavity: a case report. J Cardiothorac Surg 16:197 34247638 10.1186/s13019-021-01576-x PMC8274025

[87] Gaskin C, Helms C (2004) Lipomas, lipoma variants, and well-differentiated liposarcomas (atypical lipomas): results of MRI evaluations of 126 consecutive fatty masses. AJR Am J Roentgenol 182:733–739 14975977 10.2214/ajr.182.3.1820733

[88] Faer MJ, Burnam RE, Beck CL (1978) Transmural thoracic lipoma: demonstration by computed tomography. AJR Am J Roentgenol 130:161–163 413404 10.2214/ajr.130.1.161

[89] Chandrasekar CR, Grimer RJ, Carter SR et al (2008) Elastofibroma dorsi: an uncommon benign pseudotumor. Sarcoma 2008:756565 18382611 10.1155/2008/756565 PMC2276598

[90] Battaglia M, Vanel D, Pollastri P et al (2009) Imaging patterns in elastofibroma dorsi. Eur J Radiol 272:16–21.42 10.1016/j.ejrad.2009.05.024 19539441

[91] Ochsner JE, Sewall SA, Brooks GN, Agni R (2006) Best cases from the AFIP: elastofibroma dorsi. Radiographics 26:1873–1876 17102057 10.1148/rg.266055184

[92] Mansour J, Raptis D, Bhalla S et al (2022) Diagnostic and imaging approaches to chest wall lesions. Radiographics 42:359–378. 10.1148/rg.210095 35089819 10.1148/rg.210095

[93] Incarbone M, Pastorino U (2001) Surgical treatment of chest wall tumors. World J Surg 25:218–230 11338025 10.1007/s002680020022

