# Supplementary material for: A pictorial essay of thoracic wall diseases: multiple pathologies in the same anatomical site
Source: Insights Imaging. 2025 Sep 20;16:200. doi: 10.1186/s13244-025-02073-8 (PMC12450195; doi:10.1186/s13244-025-02073-8)

A pictorial essay of thoracic wall diseases: multiple pathologies in the same anatomical site

ELECTRONIC SUPPLEMENTARY MATERIAL

**Figure 1s.** Pectus index in a patient with pectus excavatum. In the mediastinal view, the ratio obtained between the transverse diameter of the thorax and the anteroposterior diameter of the thorax (figure a) assesses the degree of sternal dislocation within the mediastinum. CT window for lung parenchyma, confirms the deformation of the thoracic wall (figure c). Lateral MPR images (figures b and d) mediastinal and bone windows respectively, clearly show the depression of the anterior thoracic wall, with cardiac compression by the body of the sternum.


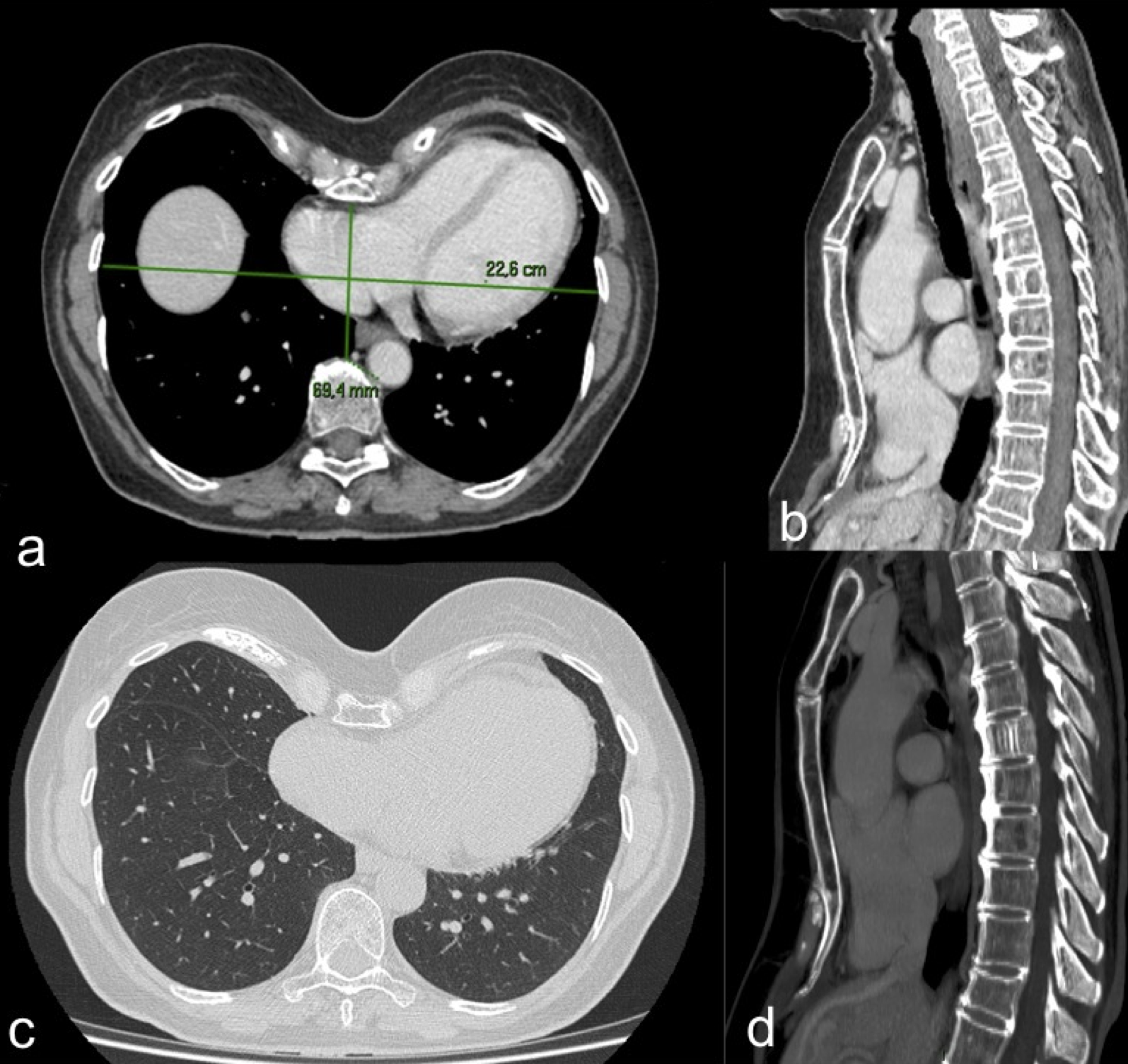


**Figure 2s.** Pectus carinatum. Sagittal MPR images after contrast-medium injection (figure a, b ) show abnormal anterior convexity of the sternum; increased distance between the sternum and thoracic ascending aorta is well depicted, Figure b (sagittal images) also demonstrates a collateral finding (collapse of a dorsal vertebra).


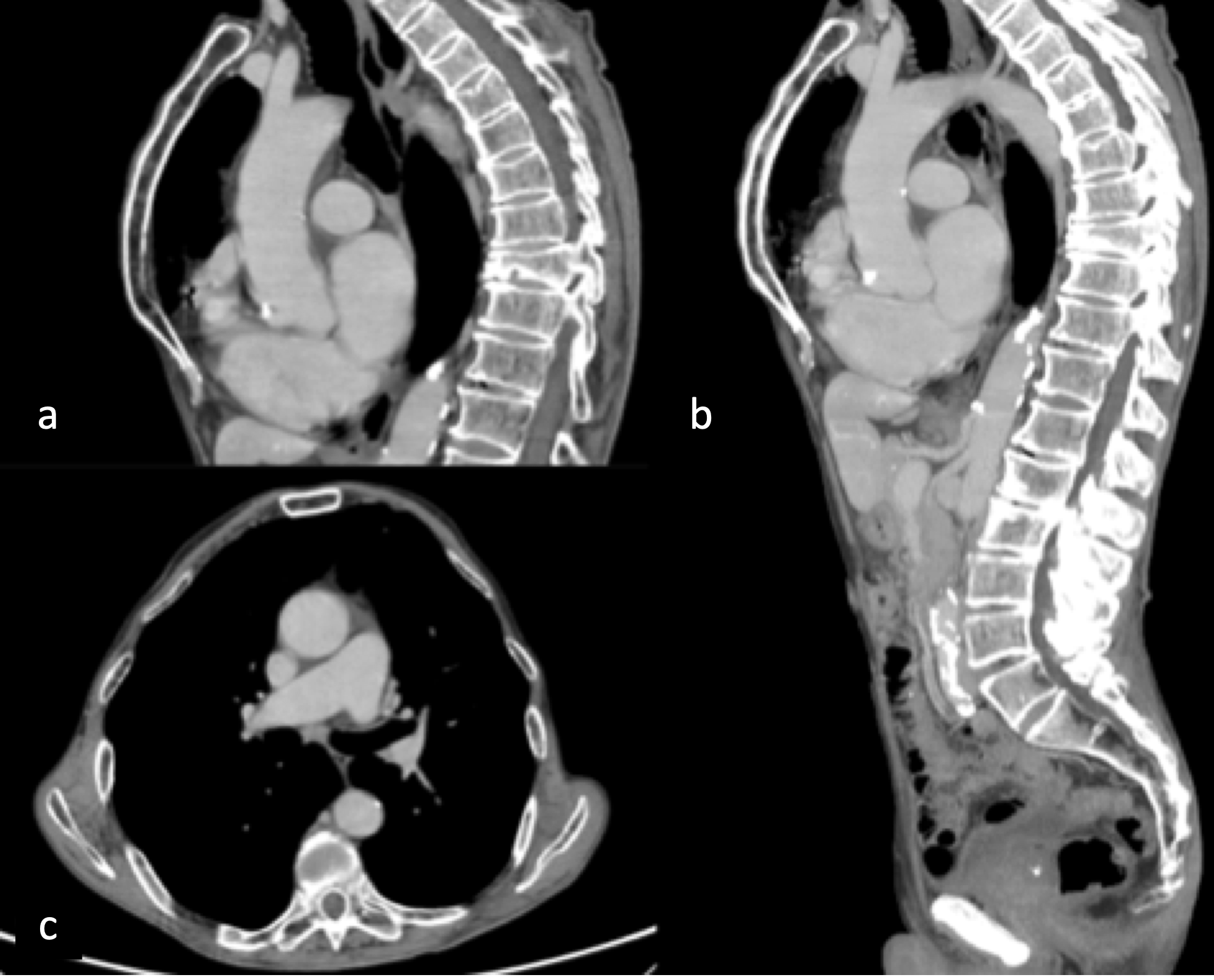


**Figure 3s.** A 27-years-old female patient with mucopolysaccharidoses syndrome. Chest X-ray shows enlargement of ribs (figure a). In a radiograph of the spine, we can see rounded vertebral bodies (Figure b). T2-weighted STIR MRI sequence shows two areas of increased signal intensity (white circle in c) are also found in the spine, at the level of Th11-Th12 due to the myelopathy as a consequence of spinal cord compression.


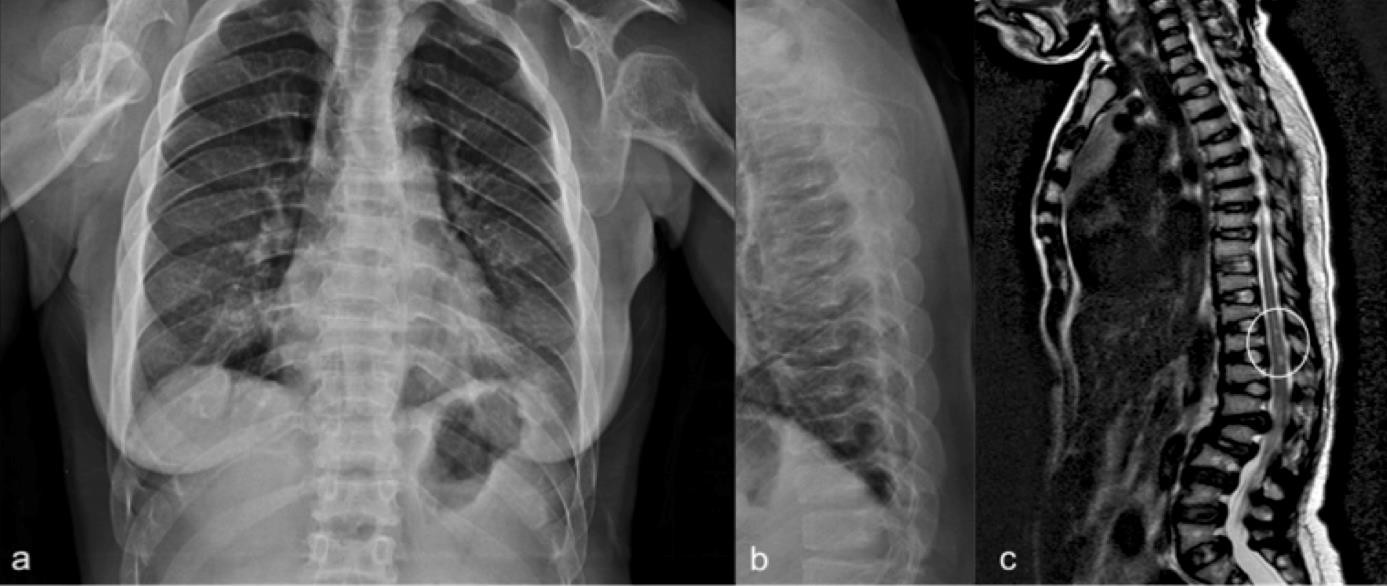


**Figure 4s.** CT imaging, mediastinal window after contrast-medium administration (venous phase, figures a and b)**.** Immunosuppressed patient (affected by myelodysplastic syndrome). Presternal abscess with fistula to the skin. The pre-sternal collection has a typical aspect with aerial content.


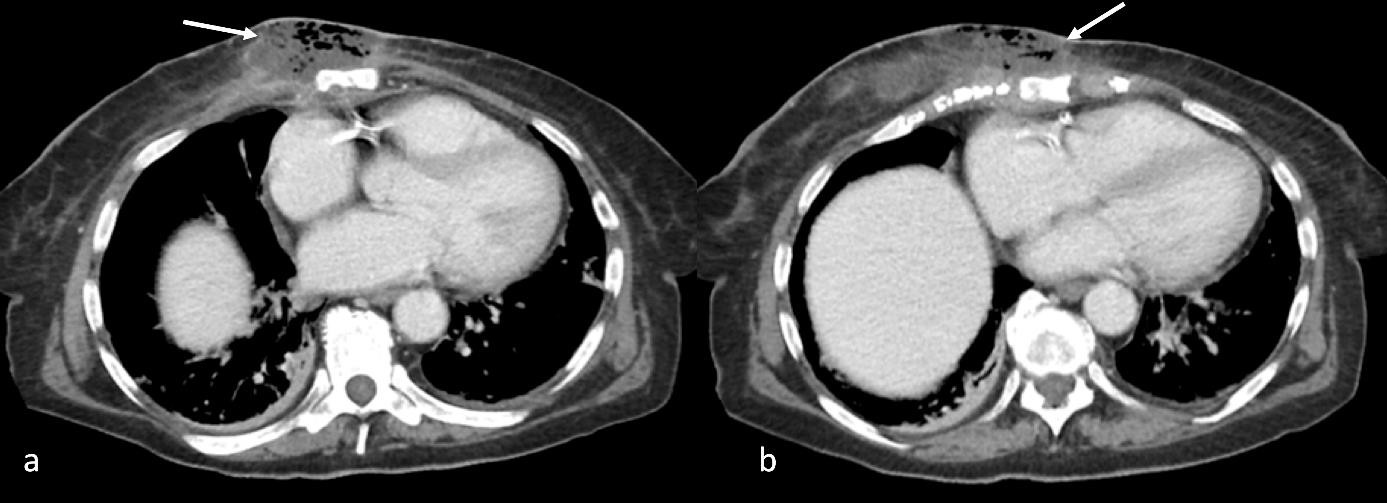


**Figure 5s.** Chest trauma, X-ray anteroposterior projection shows posterior arch displaced fractures of the III, IV, and V ribs (white arrows).


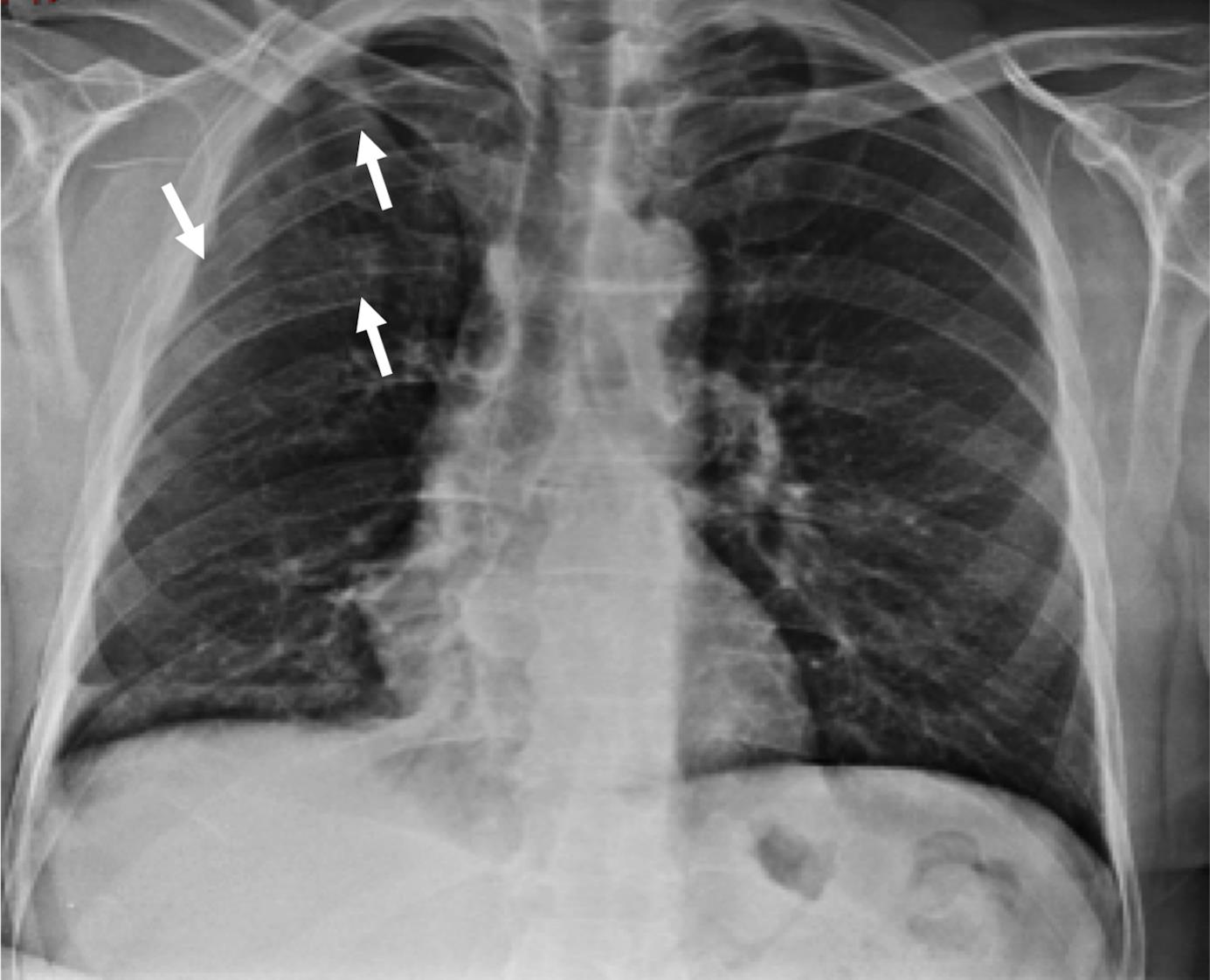


**Figure 6s.** X-ray lateral projection (figure a): fracture of the sternal manubrium with dislocation from the sternal body (white arrows). Figure b shows another case of sternal fracture: CT imaging, bone window, fracture with dislocation of the sternal manubrium.


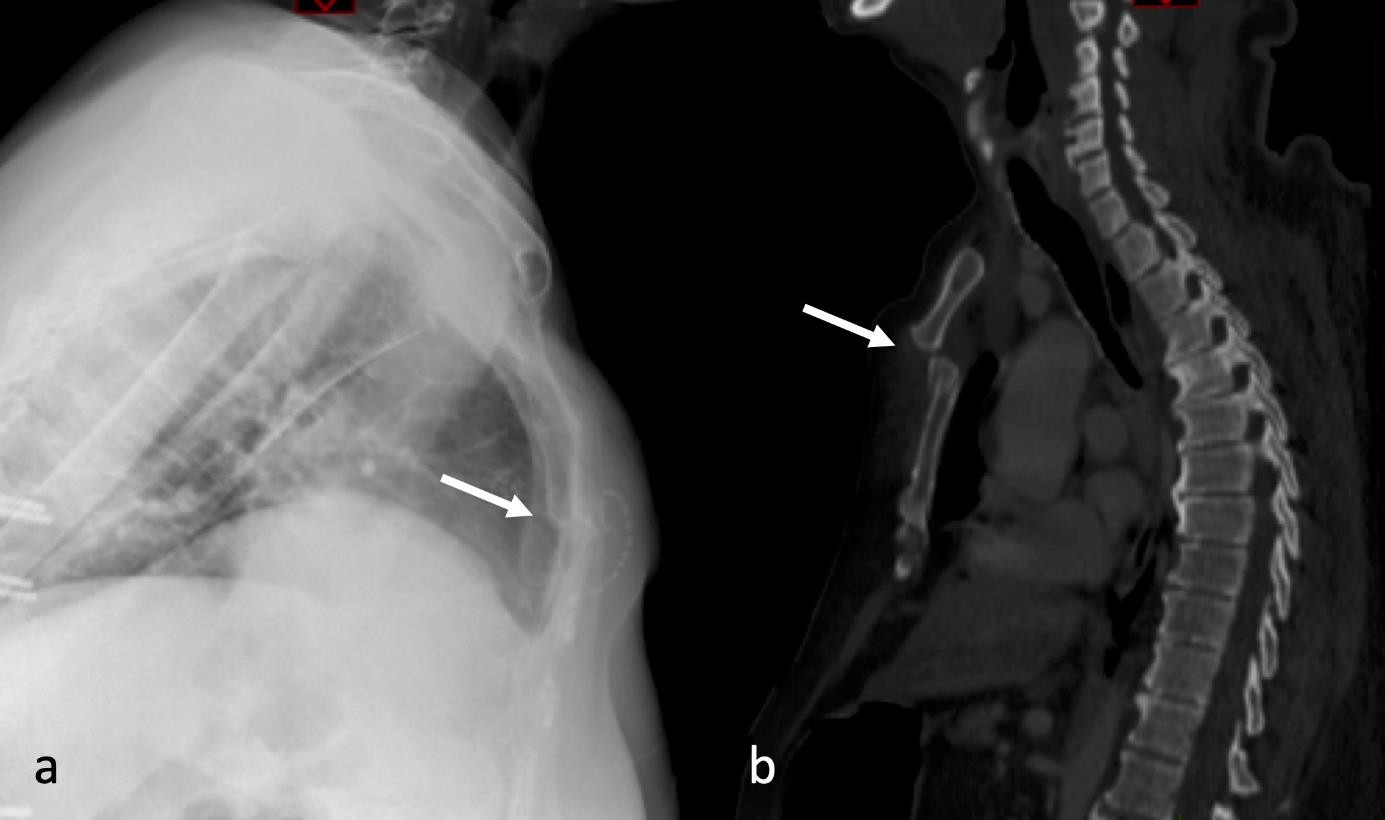


Figure 7s. CT bone reconstructions show two cases of vertebral fractures with different origins: oncological (figure a, white arrow) because of prostatic cancer, and traumatic (figure b, white circle) with rupture of the posterior wall.


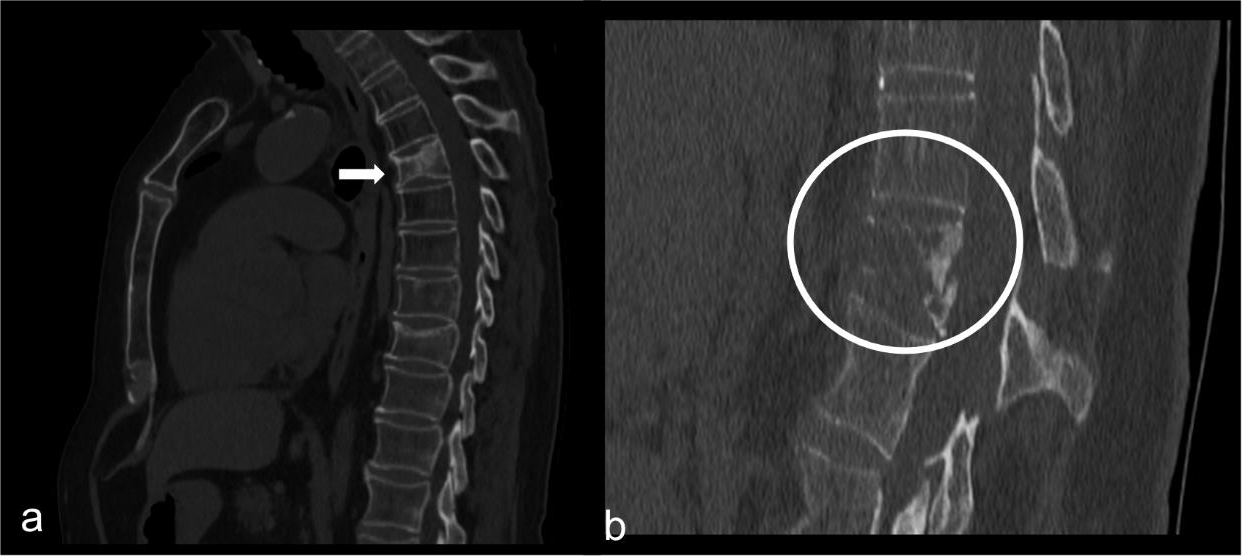


**Figure 8s.** Computed Tomography examination, evaluation after contrast-medium administration (venous phase). Neurofibromas of the chest wall in neurofibromatosis syndrome, mediastinal location. Genetic confirmation after imaging was made.


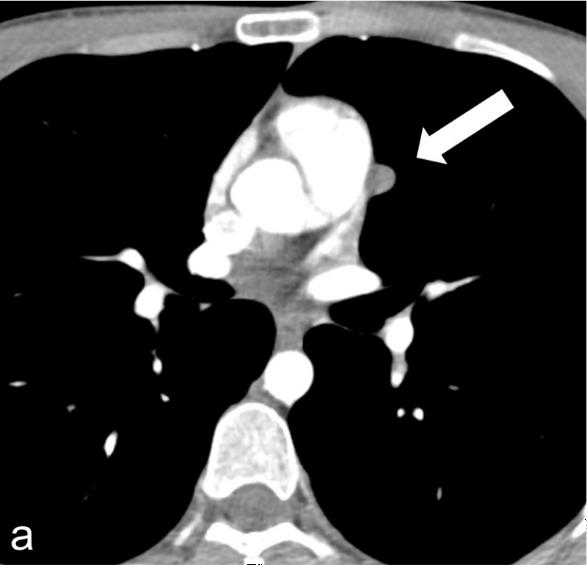


**Figure 9s.** MRI -T2 sequences with fat suppression. Figures «9s a-d» show extended neurofibroma that arises from the neck (white arrow) and reaches the upper thoracic site.


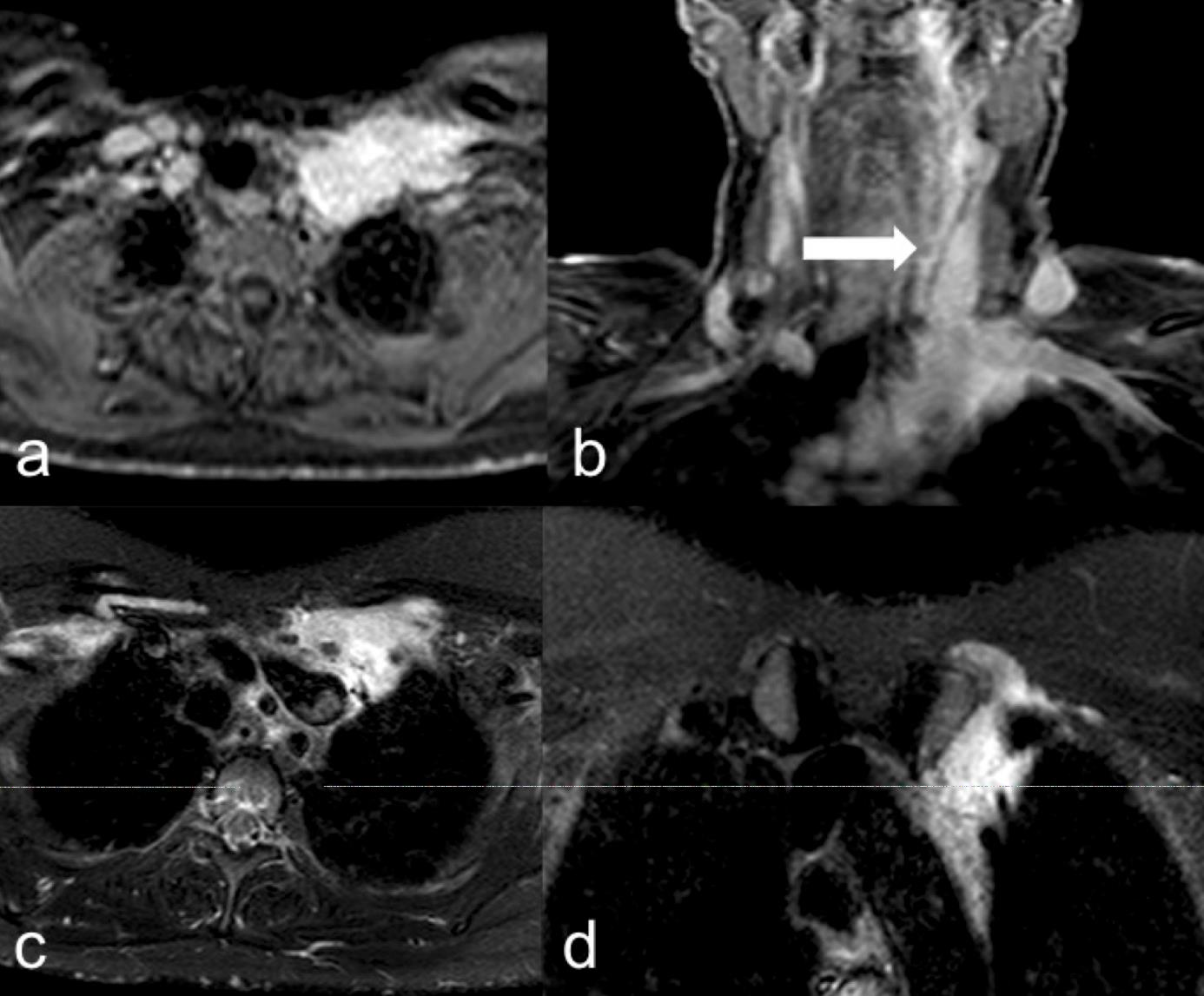


**Figure 10s**. A CT study (mediastinal window) in a 68-year-old male patient affected by neurofibromatosis syndrome type I shows: multiple cutaneous neurofibromas along the anterior chest wall (white arrows) visible in axial (figure a) and sagittal (figure b) reconstruction.


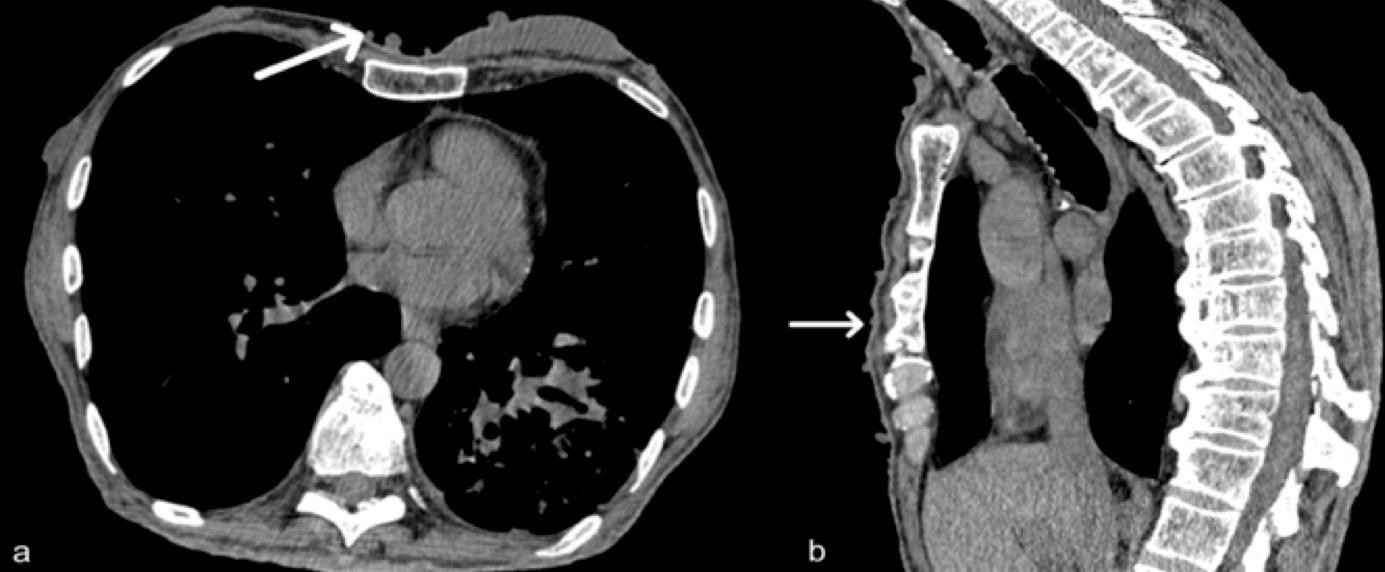


**Figure 11s.** CT imaging after contrast-medium administration, mediastinal window: incidental findings of thoracic rounded and well-defined lipoma.


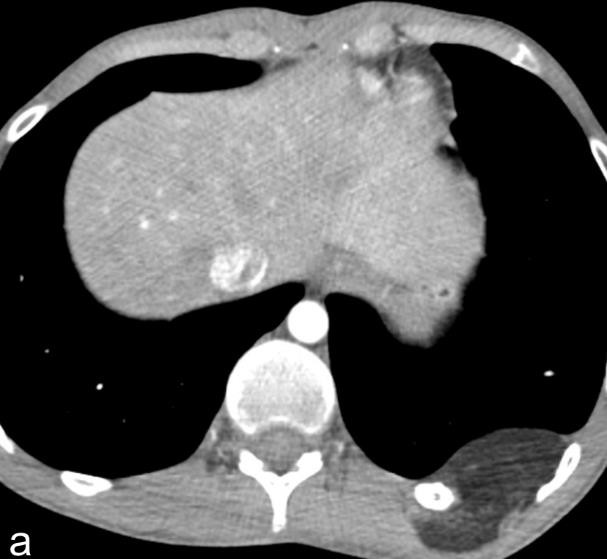


**Figure 12s.** A case of elastofibroma dorsi, typically situated in the infrascapular or subscapular region and usually bilateral (white arrows). It is a poorly defined soft-tissue mass with CT attenuation (basal phase) closely resembling adjacent skeletal muscle.


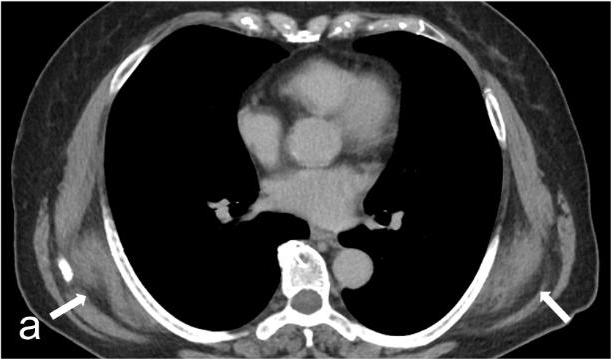

Supplement: Supplementary file 1 — ELECTRONIC SUPPLEMENTARY MATERIAL [file 13244_2025_2073_MOESM1_ESM.docx]
